# Nanoparticle-Exposure-Triggered Virus Reactivation Induces Lung Emphysema in Mice

**DOI:** 10.1021/acsnano.3c04111

**Published:** 2023-10-19

**Authors:** Lianyong Han, Verena Haefner, Youjia Yu, Bing Han, Hongyu Ren, Martin Irmler, Johannes Beckers, Qiongliang Liu, Annette Feuchtinger, Ali Oender Yildirim, Heiko Adler, Tobias Stoeger

**Affiliations:** 1 Institute of Lung Health and Immunity (LHI), Comprehensive Pneumology Center, Helmholtz Zentrum München, German Research Center for Environmental Health, 85764 Neuherberg, Germany; 2 Institute of Asthma and Allergy Prevention, Helmholtz Zentrum München, German Research Center for Environmental Health, 85764 Neuherberg, Germany; 3 Walther Straub Institute of Pharmacology and Toxicology, Ludwig-Maximilians-University Munich, 80336 Munich, Germany; 4 Department of Forensic Medicine, Nanjing Medical University, 211166 Nanjing, Jiangsu, China; 5 Laboratory of Translational Research “Stress and Immunity”, Department of Anesthesiology, LMU Hospital, Ludwig-Maximilians-University Munich, 81377 Munich, Germany; 6 Institute of Experimental Genetics, Helmholtz Zentrum München, German Research Center for Environmental Health, 85764 Neuherberg, Germany; 7 German Center for Diabetes Research (DZD), 85764 Neuherberg, Germany; 8 Technische Universität München, Chair of Experimental Genetics, 80539 Munich, Germany; 9 Research Unit Analytical Pathology, Helmholtz Zentrum München, German Research Center for Environmental Health, 85764 Neuherberg, Germany; 10 Institute of Experimental Pneumology, University Hospital, Ludwig-Maximilians University, 81377 Munich, Germany; 11 Member of the German Center of Lung Research (DZL), 81377 Munich, Germany

**Keywords:** γ-herpesvirus, virus reactivation, nanoparticles, emphysema, p38 MAPK

## Abstract

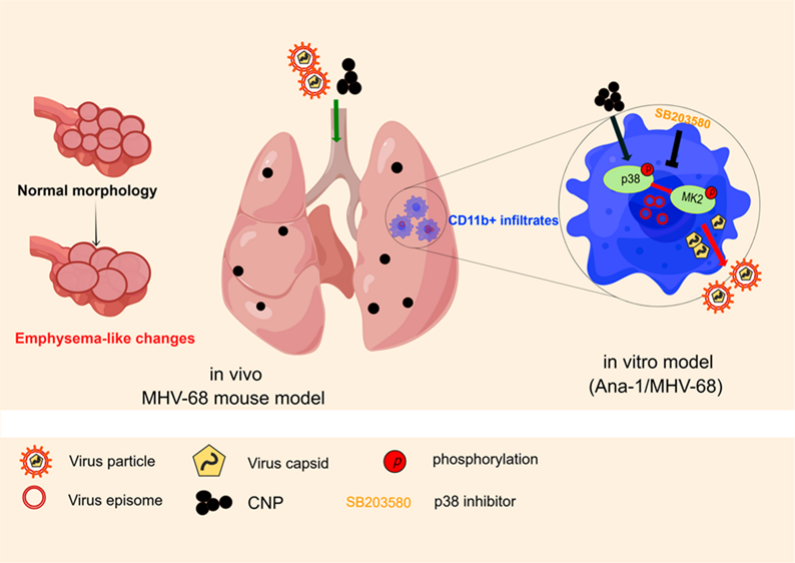

Nanoparticles (NPs) released from engineered materials or combustion processes as well as persistent herpesvirus infection are omnipresent and are associated with chronic lung diseases. Previously, we showed that pulmonary exposure of a single dose of soot-like carbonaceous NPs (CNPs) or fiber-shaped double-walled carbon nanotubes (DWCNTs) induced an increase of lytic virus protein expression in mouse lungs latently infected with murine γ-herpesvirus 68 (MHV-68), with a similar pattern to acute infection suggesting virus reactivation. Here we investigate the effects of a more relevant repeated NP exposure on lung disease development as well as herpesvirus reactivation mechanistically and suggest an avenue for therapeutic prevention. In the MHV-68 mouse model, progressive lung inflammation and emphysema-like injury were detected 1 week after repetitive CNP and DWCNT exposure. NPs reactivated the latent herpesvirus mainly in CD11b+ macrophages in the lungs. *In vitro*, in persistently MHV-68 infected bone marrow-derived macrophages, ERK1/2, JNK, and p38 MAPK were rapidly activated after CNP and DWCNT exposure, followed by viral gene expression and increased viral titer but without generating a pro-inflammatory signature. Pharmacological inhibition of p38 activation abrogated CNP- but not DWCNT-triggered virus reactivation *in vitro*, and inhibitor pretreatment of latently infected mice attenuated CNP-exposure-induced pulmonary MHV-68 reactivation. Our findings suggest a crucial contribution of particle-exposure-triggered herpesvirus reactivation for nanomaterial exposure or air pollution related lung emphysema development, and pharmacological p38 inhibition might serve as a protective target to alleviate air pollution related chronic lung disease exacerbations. Because of the required precondition of latent infection described here, the use of single hit models might have severe limitations when assessing the respiratory toxicity of nanoparticle exposure.

## Introduction

Applications and demand for new nanomaterials are mounting; accordingly, nanostructure materials are developed, produced, and used ever-increasingly in our modern life. However, concerns have been raised about the release of nanoparticles from nanoenabled, engineered advanced materials into the environment.^1^ Of particular concern are respiratory health effects related to particle inhalation, thereby generating an urgent need for adequate risk assessment with respective testing tools and management throughout the entire life cycle of such products. In anthropogenic air pollution, it has been recognized as a global problem of great concern to human health, and the WHO estimates that around 7 million people die due to poor air quality per year. Environmental particles, especially carbonaceous particulate matter (PM) derived from combustion processes, are one of the major sources of air pollutants,^2^ and long-term exposure to air pollution has been associated with increased risk of chronic lung diseases (CLDs), particularly chronic obstructive pulmonary disease (COPD).^3−5^ Inhalation of soot-like carbonaceous nanoparticles (CNPs) is well-known to induce acute inflammatory responses, both locally in lungs and systemically in the cardiovascular system of exposed people,^6^ as well as in toxicological animal studies.^7,8^ Due to their fiber-shape and asbestos homology, some specimen of engineered carbon nanotubes (CNTs) can induce persistent inflammation and even chronic lung injury.^9,10^ Based on the central role of inflammation, pulmonary nanoparticle exposure was reported to contribute to the development of CLDs, like asthma, lung fibrosis, and even cancer and in particular COPD.^11−16^

Also viral infection, another unavoidable environmental challenge, is suspected to contribute to the development and exacerbation of CLDs.^17−19^ Here, herpesviruses are among the most prevalent and persistent human pathogens,^20^ including the γ-herpesviruses Epstein–Barr virus (EBV) and Kaposi’s sarcoma-associated herpesvirus (KSHV/HHV8). These specialized DNA viruses share a biphasic lifecycle characterized by a lytic phase during primary infection, followed by latency until reactivation. During primary, acute infection, several cell types, particularly epithelial barrier cells, get infected, resulting in lytic cell death and acute tissue damage. After the acute infection γ-herpesviruses usually establish latency in macrophages, B lymphocytes, and dendritic cells (DCs),^21,22^ and once the host gets hit by certain challenges, latent viruses can get reactivated and re-enter the lytic cycle again, producing infectious virus particles similar to that during acute infection.^23^ Murine γ-herpesvirus 68 (MHV-68) infected mice serve as a widely used animal model to study the pathogenesis of γ-herpesvirus infections. MHV-68 open reading frames (ORFs) 50 and 73 encode viral proteins that are particularly important for the viral life cycle. ORF50 encodes the replication and transcriptional activator (RTA) and plays an essential role in virus replication as well as in reactivation from latency.^24,25^ As a major regulator of viral gene transcription, RTA (ORF50) thus determines the life cycle of MHV-68. MHV-68 ORF73 in contrast is required for viral episome maintenance and is expressed throughout both the lytic and latent phases. Assessing the balance between ORF50 and ORF73 expression levels can thus be used to monitor lytic versus latent states of the virus life cycle. γ-Herpesviruses are omnipresent cofounding factors for many diseases and levels of endemic infection are very high, with EBV being carried by more than 90% of the population.^26^ In this context, EBV virus load has been implicated in the progression of COPD, and elevated levels of EBV DNA have been detected in about 50% of COPD exacerbation cases or patients with stable COPD, in comparison to only 6% in unaffected smokers.^27^ In addition, high γ-herpesvirus DNA levels were reported for patients suffering bronchiectasis^28^ and pulmonary fibrosis.^29,30^ A recent meta-analysis also suggests that persistent or chronic EBV and HHV8 infections increase the risk of idiopathic pulmonary fibrosis (IPF) development.^31^

In view of the omnipresent burden of environmental and engineered nanoparticle exposure and persistent herpesvirus infection, we had previously shown in lungs of latent MHV-68 infected mice that a single pulmonary exposure to CNPs or double-walled carbon nanotubes (DWCNTs) induced an increase of lytic viral protein expression, with similar patterns of metabolite and gene expression changes as observed during acute infection, thereby indicating pulmonary virus reactivation. Likewise, we showed that exposure of human cells latently infected with Epstein–Barr virus to CNPs also induced virus production.^32^ However, to mimic an environmental exposure scenario, not only a single exposure but also multiple hits with high particle burden need to be included into our experimental model to study herpesvirus reactivation and related effects on lung injury.

Mechanistically, mitogen-activated protein kinase (MAPK) signaling, an important stress response pathway, is considered to facilitate γ-herpesvirus lytic infection and replication,^33−35^ but to what extend MAPK signaling is also required for virus reactivation is not yet clear. Nevertheless, in a lymphoma cell model chemically induced reactivation of latent KSHV was shown to be MAPK dependent,^36^ and the MAPK pathway is well-known to be activated by many particle–cell interactions. For instance, amorphous silica nanoparticles can rapidly activate MAPK signaling in exposed RAW264.7 macrophages, via triggering a pro-inflammatory response.^37^ In this context, also cellular oxidative stress and the formation of reactive oxygen species (ROS), a well-known activator of MAPK signaling, are widely reported to be triggered by nanoparticle–cell interaction, and the oxidative potential of carbon based nanoparticles is closely associated with their potency to induce pulmonary inflammation.^38^ Ye et al. described hydrogen peroxide (H_2_O_2_) mediated KSHV reactivation via p38, ERK1/2, and JNK pathways,^39^ and ROS scavenging via *N*-acetyl-l-cysteine (NAC) effectively inhibited MAPK signaling and KSHV lytic infection *in vitro* and *in vivo*. On this basis, we hypothesized that nanoparticle exposure reactivates MHV-68 via a ROS–MAPK signaling pathway.

Combining *in vivo* and *in vitro* models, we provide here evidence that (i) repeated NP exposure can caused interstitial lung inflammation and notably progressive epithelial injury with emphysema in the MHV-68 animal model, (ii) CD11b+ macrophages execute the virus reactivation in the lung and in MHV-68 pseudo-latently infected murine macrophages CNPs triggered MHV-68 reactivation via a p38 MAPK dependent signaling pathway, and (iii) pharmacological inhibition of p38 could attenuate CNP-induced MHV-68 reactivation in mouse lungs *in vivo*. Targeting p38 might thus serve as a protective mechanism to alleviate ambient particle exposure related lung damage and emphysema development. Fiber toxicity, as related to inhalation of CNTs, however, seems more complex and does not depend on p38 activation alone.

## Results

### NPs Caused Emphysema-like Changes in Mouse Lungs Latently Infected with MHV-68

**Figure 1:**
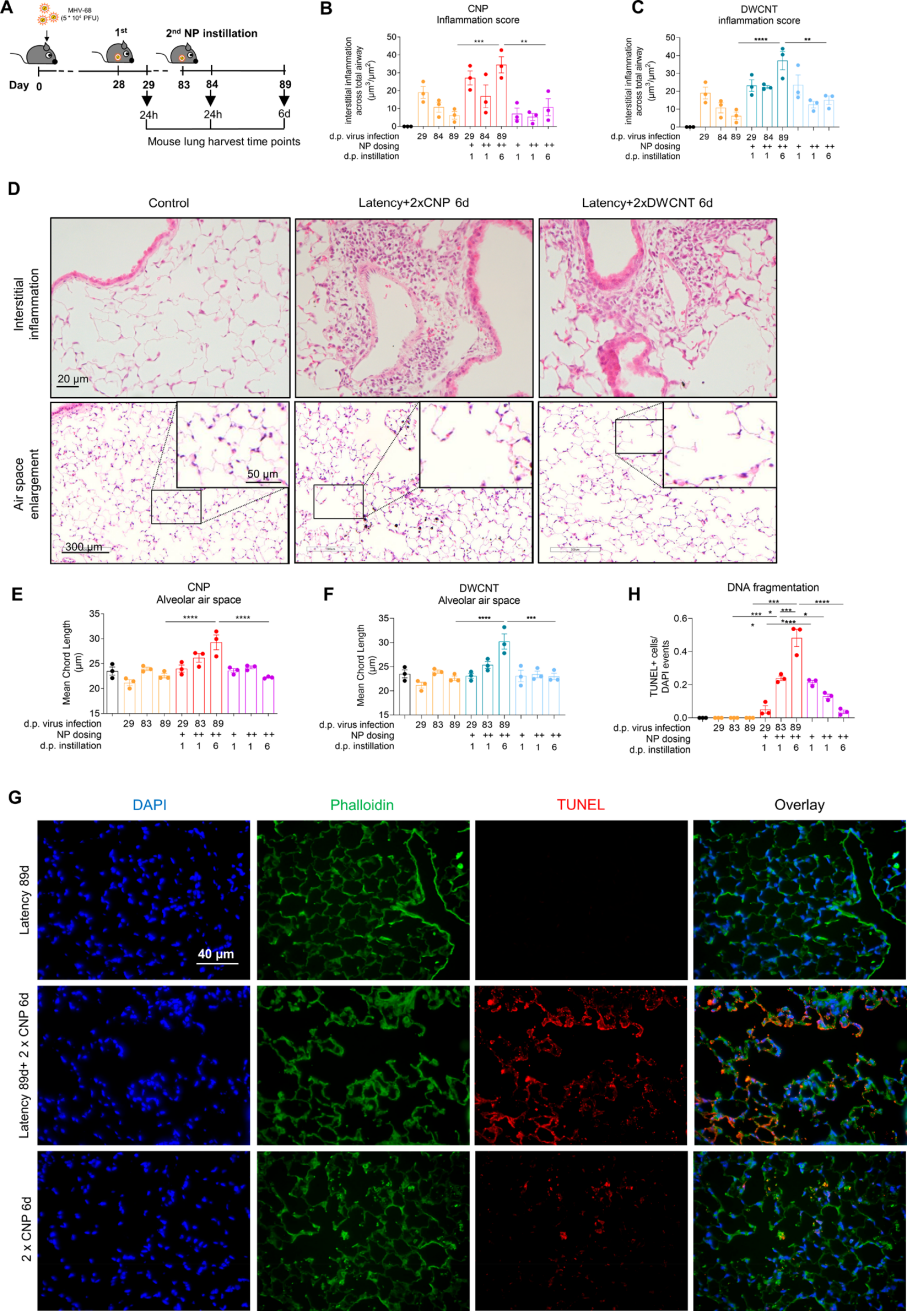
Repeated NP exposure of MHV-68 infected mice increases alveolar air space and lung tissue inflammation. (A) Schematic illustration of the animal experiment. C57BL/6 mice were intranasally infected with 5 × 10^4^ PFU of MHV-68 and, after establishment of latency, exposed to 50 μg of NPs (CNPs or DWCNTs) by intratracheal instillation at days 28 and 83 postinfection. 24 h after the first and second NP (CNP or DWCNT) exposure and additionally 6 days after the second NP instillation, mouse lungs were harvested for histological analysis of structural and inflammatory changes in the lung tissue. (B) Quantification of inflammatory cell accumulation in H&E stained slides using the CAST method shows increased levels of tissue inflammation in lungs of mice 89 days postinfection and 6 days after the second CNP instillation (*N* = 3). (C) Quantification of inflammatory cell accumulation in H&E stained slides using the CAST method shows increased levels of tissue inflammation in lungs of mice 89 days postinfection and 6 days after the second DWCNT instillation (*N* = 3). (D) H&E stained lung tissues indicate interstitial inflammation and alveolar wall loss and increased air space in lung tissues of mice 89 days after MHV-68 infection and 6 days after second CNP and second DWCNT instillation. Upper scale bar: 20 μm; lower scale bars: 300 and 50 μm. (E) Quantification of the morphometric changes measuring mean chord length (MCL) using the CAST method verifies significant alveolar air space enlargement in lungs of mice 89 days postinfection and 6 days after the second CNP instillation (*N* = 3). (F) Quantification of the morphometric changes measuring mean chord length (MCL) using the CAST method verifies significant alveolar air space enlargement in lungs of mice 89 days postinfection and 6 days after the second DWCNT instillation (*N* = 3). (G) Representative immunofluorescence labeling of DNA fragmentation (TUNEL) in lung tissue indicates increased DNA fragmentation and cell death in lung tissue of mice 89 days after MHV-68 infection and 6 days after second CNP instillation compared to latency (day 90 after infection) or CNP (day 6 after second instillation) only. DAPI: blue, phalloidin: green, TUNEL: red. Scale bar: 40 μm. (H) Quantification of the TUNEL positive cells relative to DAPI events verifies a significant increase of DNA fragmentation in repeated CNP instillation in mice latently infected with MHV-86 (*N* = 3). Values were shown as mean ± SEM. Data were analyzed by Ordinary one-way ANOVA and Tukey’s multiple comparisons test; “*” indicates statistically significant difference between displayed groups. * indicates *P* value < 0.05. ** indicates *P* value < 0.01. *** indicates *P* value <0.001. **** indicates *P* value < 0.0001.

During occupational or environmental exposure scenarios, people may experience repeated episodes of high particle burden, eventually causing additive effects to the lungs. Here, we investigated the effect of repeated carbonaceous NP exposure, either CNPs or DWCNTs, on lung inflammation and the potential development of epithelial damage and chronic lung injury. C57BL/6 mice were intranasally infected with MHV-68 (5 × 10^4^ PFU), and after establishment of latency, mice were exposed to 50 μg of NPs by intratracheal instillation at days 28 and 83 postinfection. Twenty-four hours after the first and second NP exposures and additionally 6 days after the second NP instillation, mouse lungs were harvested to investigate acute and progressive pathological changes (Figure 1A). The area of inflammatory cells recruited to the interstitium was quantified relative to the airways and vessels to assess the level of interstitial inflammation. Even though both NPs caused a similar level of MHV-68 reactivation investigated by lytic virus protein staining in lung tissue (Figure S1A), a significantly elevated level of interstitial inflammation in lungs of mice latently infected for 89 days was detected 6 days after the second NP instillation (Figure 1B,C), while no comparable inflammation was observed for mice only infected or only NP treated 2 times (see Figure S1F for all conditions). This effect at day 6 after repeated NP exposure was associated with elevated BAL protein (Figure S1B,C) and IgM levels (Figure S1D,E), indicating alveolar epithelial barrier disruption. A closer look into the peripheral lung structure, as shown in Figure 1D, revealed an emphysema-like loss of alveolar walls in mice infected for 89 days and 6 days after repeated NP exposure (see Figure S1G for all conditions). Mean chord length (MCL) quantification confirmed significant alveolar air space enlargement (Figure 1E,F). Assessing cell death related DNA fragmentation via TUNEL showed dramatically increased signals in the alveolar epithelium of mice infected for 89 days and 6 days after the repeated CNP exposure (Figure 1G). Quantification of DNA fragmentation confirmed significantly increased cell death in lungs of virus infected mice repeatedly CNP treated; noteworthy cell death signals increased even more until day 6 after the repeated CNP exposure, a pattern not observed for only CNP exposed, not infected mice (Figure 1H).

Taken together, repeated NP exposure of MHV-68 infected mice caused interstitial lung inflammation, injury with epithelial cell death, and emphysema-like changes, progressively increasing during the 6 days after the last exposure.

### NP-Induced Herpesvirus Reactivation Localizes to CD11b+ Macrophage-like Infiltrates

**Figure 2:**
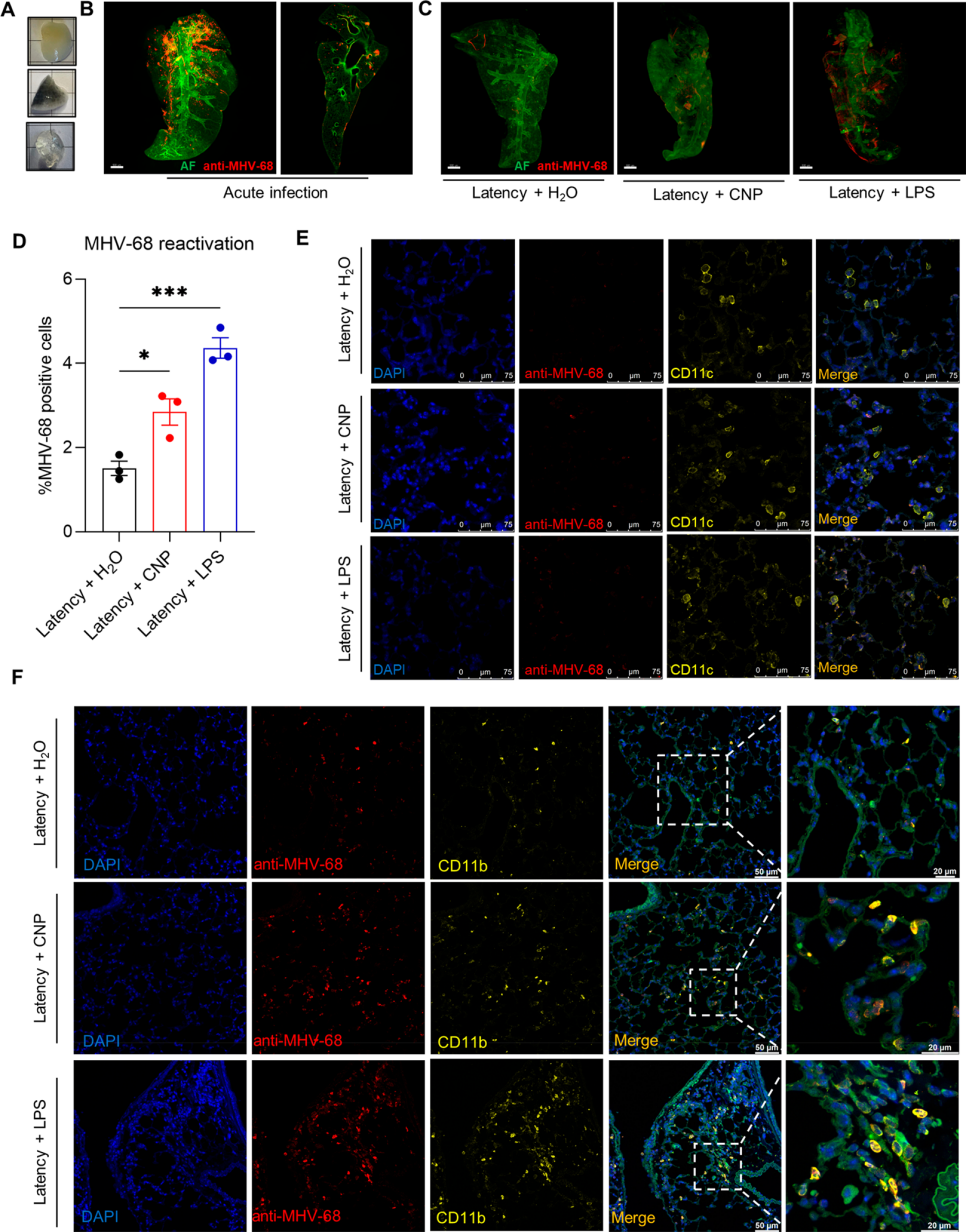
MHV-68 reactivation induced by CNPs mainly localizes to CD11b+ macrophage-like infiltrates in the lungs of mice. (A) Whole-mount images show lung lobes used for immunofluorescent (IF) labeling of latently infected (d28 postinfection) mice, before (upper and middle) and after tissue optically clearing (lower), from sham exposed mice (upper and lower), or 24 h after CNP exposure (middle). Each background square is 0.5 × 0.5 cm^2^. (B) Overview of IF detection of MHV-68 lytic protein expression during acute infection (d6 after infection). Lung lobes were incubated with rabbit anti-MHV-68 serum and stained by fluorescence-labeled anti-rabbit secondary antibody (red). Autofluorescence of lung tissue (green) illustrates the morphological structure of the bronchiolar tree. Imaging was performed with a Light-sheet fluorescence microscope (LSFM) and reconstituted with Imaris software (version 9.3.1). Magnification: 0.63×; scale bar: 500 μm. The vertical snapshot image demonstrates that MHV-68 predominately infected the epithelium. Scale bar: 1000 μm. (C) Overview of MHV-68 lytic virus protein expression during latency and reactivation induced by CNP (50 μg, 24 h) or the LPS positive control (0.1 μg, 24 h) in mouse lung lobes. Scale bar: 500 μm. (D) Quantification of the percentage of MHV-68 lytic protein expressing lung cells detected by IF-histology demonstrates significant MHV-68 reactivation upon CNP and LPS exposure. IF stained lung slides were scanned with Zeiss Axio Scan.Z1 (ZEISS, Germany), and quantification was performed with Definiens Enterprise Image Intelligence Suite (Definiens). Values are shown as mean ± SEM (*N* = 3). One-way ANOVA followed by Tukey’s multiple comparisons test was used for statistical analysis. *P < 0.05. ***P < 0.001. (E) IF double staining identifies that MHV-68 lytic protein (red) only rarely localized to CD11c positive resident lung macrophages (CD11c: yellow; Phalloidin 488: green; DAPI: blue). Scale bar: 75 μm. (F) MHV-68 lytic protein expression is predominately induced by CNPs and LPS in CD11b positive macrophage like infiltrates (anti-MHV-68 serum: red; CD11b: yellow; Phalloidin 488: green; DAPI: blue). Images were taken with a confocal fluorescence microscope (Leica, TSC-SP5-II, Wetzlar, Germany). Scale bar: 50 or 20 μm as indicated.

To identify the cell type in which the virus reactivation occurred, we performed immunofluorescence and advanced microscopy analysis and focused on CNP exposed lungs since the reactivation pattern triggered by DWCNTs matched very well that by CNPs (Figure S1A). Light-sheet fluorescence microscopy (LSFM) provided an overview of MHV-68 infection and reactivation throughout the lungs by immunostaining of entire lobes with polyclonal rabbit serum directed against lytic proteins of MHV-68, followed by optical tissue clearing (Figure 2A). During the phase of acute infection (day 6 after infection), abundant lytic MHV-68 protein expression was detected in central bronchiolar patches but also reaching to distal areas of the lung (Figure 2B). While no MHV-68 protein was detected during latency (28 days after infection), CNPs (50 μg/mouse) induced more diffuse lytic virus protein expression 24 h after exposure (Figure 2C). LPS (lipopolysaccharide; 0.1 μg/mouse) served as positive control and generated higher levels of reactivation, suggesting a link of the virus response to inflammatory cell recruitment. Quantitative analysis revealed an approximately 2-fold increase of reactivating cell numbers induced by CNPs and a 3-fold increase induced by LPS exposure compared to the sham control group (Figure 2D).

Since the immunohistology suggested that MHV-68 reactivation occurred mainly in lung areas exhibiting mononuclear cell infiltrations but not in CNP particle agglomerate laden alveolar macrophages (Figure S2A), we performed double staining of anti-MHV-68 immunofluorescence together with the monocyte and macrophage markers CD11b and CD11c. The integrin alpha M CD11b is predominantly expressed in monocytes, monocyte-derived macrophages, and granulocytes and served as marker for recruited monocytes and macrophages of the interstitial lung infiltrates. The integrin alpha X CD11c, in contrast, is known for its expression in dendritic cells, alveolar macrophages, and some interstitial macrophages, where highest expression marks resident alveolar macrophages.^40^ On the alveolar surface, patrolling alveolar macrophages are essential for rapid particle phagocytosis, and accordingly, a large fraction of CD11c positive cells feature internalized particle agglomerates 24 h after CNP exposure **(**Figure S2A). However, as shown in Figure 2E, only very few (about 0.3%, Figure S2C) CD11c positive cells showed detectable MHV-68 lytic protein expression, and their number did not change upon particle treatment, indicating that resident alveolar macrophages are not the prime MHV-68 reactivating cells. Instead, the lytic virus protein predominantly localized to CD11b positive cells, which were frequently seen in regions with mononuclear infiltrations and only rarely in the alveolar space (Figure 2F; Figure S2). These CD11b positive cells, however, did not show recognizable CNP agglomerates (Figure S2B). Importantly the fraction of MHV-68 lytic protein expressing cells increased upon CNP treatment from 1% during latency to 2% 24 h after CNP exposure and 4% after LPS treatment, reflecting the lytic protein increase observed in total (Figure 2D). Additional markers for monocyte-derived macrophages but not granulocytes such as AIF-1 (allograft inflammatory factor 1) or GPNMB (glycoprotein nmb) showed patterns corresponding to CD11b (data not shown). No colocalization of the lytic protein was observed with B-cell and T-cell markers (B220 and CD3, data not shown). Because of the association of virus reactivation with alveolar wall destruction, lytic protein expression induced by CNP exposure was also investigated in CD11b or CD11c positive cells recovered by bronchoalveolar lavage (BAL). While lymphocytes accumulated in the airspace during latency, the total BAL cell number increased by CNP and LPS was mainly attributed to the airspace accumulation of neutrophils (Figure S3A). However, only rarely anti-MHV-68 positive cells were detected in BAL, among which there was virtually no anti-MHV-68 colocalization with CD11c positive alveolar macrophages (Figure S3B) but occasionally with very rarely detected CD11b positive macrophage like cells (Figure S3C,D).

Taken together, pulmonary delivered CNP particles get rapidly phagocytosed by alveolar macrophages, but NP-exposure-triggered MHV-68 reactivation in mouse lungs happened predominantly in CD11b positive macrophage-like infiltrates of the interstitium and less in the airspace.

### Mechanistic Study of NP-Triggered Herpesvirus Reactivation in Bone Marrow-Derived Macrophages (BMDMs)

**Figure 3:**
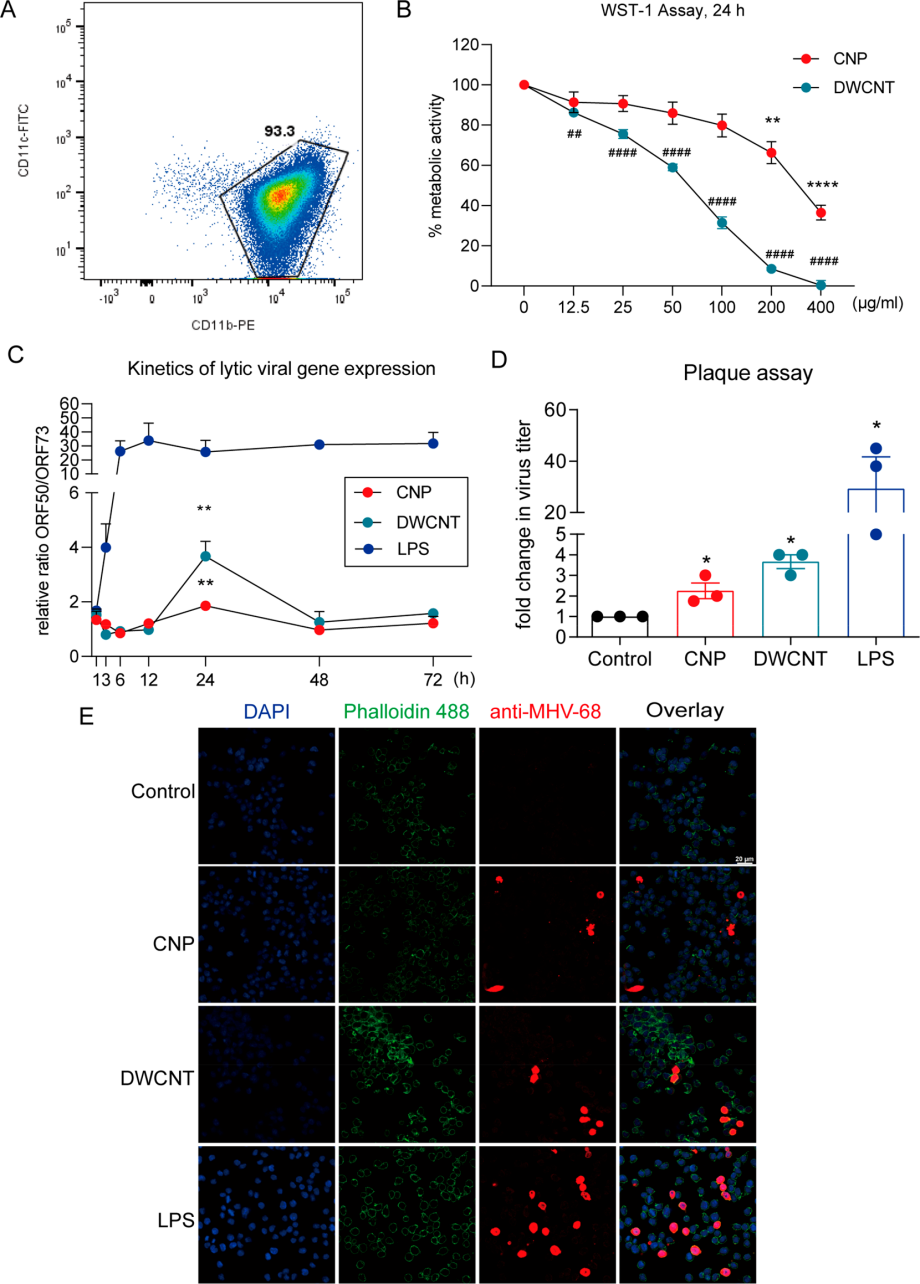
CNP- and DWCNT-triggered MHV-68 reactivation in pseudo-latently infected Ana-1 (Ana-1/MHV-68) cells. (A) Flow cytometry of Ana-1 cells demonstrates high CD11b versus low CD11c expression, rendering the bone marrow derived macrophage cell line an appropriate model for mechanistic studies of MHV-68 reactivation. (B) Cell viability (WST-1) analysis shows dose-dependent decreases of Ana-1 cells 24 h after CNP or DWCNT exposure and justifies the selected dose of 50 μg/mL as appropriate to study virus reactivation at low cytotoxicity (*N* = 3). Values are shown as mean ± SEM. Data were analyzed by Ordinary one-way ANOVA and Tukey’s multiple comparisons test; “*” indicates statistically significant difference between “CNP” and “control”, whereas “#” indicates statistically significant difference between “DWCNT” and “control”. **P < 0.01. ****P < 0.0001. ^##^*P* < 0.01. ^####^*P* < 0.0001. (C) Kinetics of lytic viral gene expression, triggered by 50 μg/mL CNP and DWCNT in Ana-1/MHV-68 cells, was monitored by qPCR, indicating a peak of lytic gene expression around 24 h for both NPs, whereas the LPS (1 μg/mL) response was considerably faster. RNA was isolated after 1–72 h of treatment, and the ratio of ORF50 expression (encoding the replication and transcription activator gene, RTA), specific for the lytic phase, versus ORF73 expression (required for viral episome maintenance), expressed throughout the lytic and latent phase, was quantified by qPCR (*N* = 4). *Rpl8* was used as the housekeeping gene. All values in the figure are shown as mean ± SEM. Data were analyzed by Student’s *t* test; “*” indicates statistically significant difference versus “control”. *P < 0.05, **P < 0.01. (D) Plaque assay from Ana-1/MHV-68 cell supernatants, performed 72 h after NP (50 μg/mL) or LPS (1 μg/mL) exposure, demonstrates significant increases in infectious virus titers (*N* = 3). All values in the figure are shown as mean ± SEM. Data were analyzed by Student’s *t* test; “*” indicates statistically significant difference versus “control”. *P < 0.05, **P < 0.01. (E) Similarly, IF staining with anti-MHV-68 serum showed an increase of lytic virus protein expression in Ana-1/MHV-68 cells 24 h after NP (50 μg/mL) or LPS (1 μg/mL) treatment, confirming the applicability of the cell-based reactivation model (anti-MHV-68 lytic protein: red; Phalloidin 488: green; DAPI: blue).

To study the mechanism underlying NP-triggered herpesvirus reactivation, we used the previously described *in vitro* model of a “pseudo-latently” MHV-68 infected murine bone marrow-derived macrophage (BMDM) cell line (Ana-1/MHV-68).^32^ Flow cytometry analysis confirmed Ana-1 cells as CD11b but not CD11c expressing macrophages, thereby mimicking the *in vivo* situation (Figure 3A). To identify the proper dose and timing conditions for mechanistic studies with the CNP- and DWCNT-treated cell line, we selected a dose of 50 μg/mL, maintaining 80% and 60% cell viability, respectively (Figure 3B). MHV-68 ORF50 encodes the replication and transcriptional activator (RTA) and plays an essential role in virus replication as well as reactivation from latency.^24,25^ RTA governs viral gene transcription; thus, ORF50 determines the life cycle of MHV-68. ORF73 is required for viral episome maintenance and is expressed throughout both the lytic and latent phase. Thus, we use the ratio of ORF50 to ORF73 as an indicator of the initiation of lytic viral gene expression, i.e., reactivation from latency. Kinetic investigation for lytic viral gene expression revealed that both CNPs and DWCNTs induced the most significant increases of lytic viral gene expression after 24 h (Figure 3C), indicating the activation of RTA and the shift of virus life cycle from latency to reactivation. Furthermore, the production of infectious virus particles was most significant 72 h after particle treatment as determined by plaque assay (Figure 3D). In a similar manner, immunofluorescence staining showed that both NPs induced lytic virus protein expression within 24 h (Figure 3E).

### Oxidative Stress Response Is Not Required for NP-Induced Herpesvirus Reactivation

**Figure 4:**
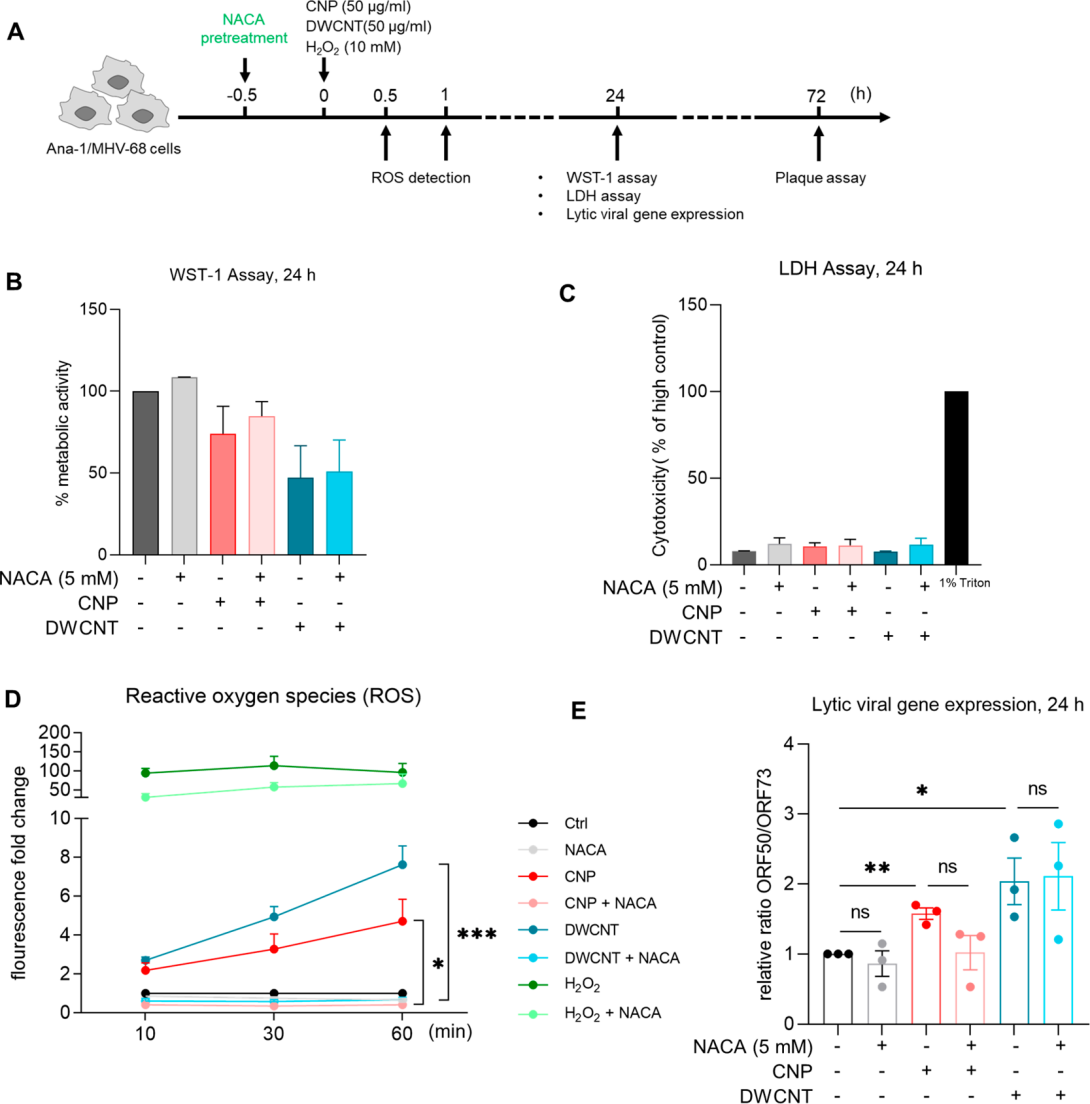
NPs trigger intracellular reactive oxygen species (ROS) production, and *N*-acetylcysteine amide (NACA) efficiently scavenges ROS but does not block MHV-68 reactivation. (A) Dichlorodihydrofluorescein diacetate (DCFH-DA) was used to investigate intracellular ROS produced by NP exposure, and *N*-acetylcysteine amide (NACA) was used to study the involvement of oxidative stress in MHV-68 reactivation induced by NPs. Briefly, Ana-1/MHV-68 cells were pretreated with NACA (5 mM) 30 min prior to treatment with NPs (50 μg/mL) or LPS (1 μg/mL). An equal amount of medium applied to cells was included as control. WST-1 assay and LDH assay were performed after 24 h and are shown in panels B and C. Two independent experiments were performed. (D) CNP- and DWCNT-triggered ROS production and efficient scavenging by NACA. DCFH-DA (15 μM) was used to measure ROS 10, 30, and 60 min after exposure using microplate reader with excitation and emission wavelength at 485 and 535 nm, respectively. (E) NACA pretreatment did not block lytic viral gene expression induced by CNP and DWCNT after 24 h in Ana-1/MHV-68 cells. Three or four independent experiments were performed and included for statistical analysis. Data were analyzed by Student’s *t* test; “*” indicates statistically significant difference versus “control”. *P < 0.05. “ns” indicates no significance.

Since NP-induced oxidative stress is considered as a canonical trigger of toxicological cellular responses such as inflammation^38,41^ and oxidative stress is also known to elicit herpesvirus reactivation,^39,42^ we considered the NP–Ana-1 cell interaction related oxidative stress response a promising candidate pathway to be investigated. The workflow is shown in Figure 4A. Intracellular levels of reactive oxygen species (ROS) were detected with the dichlorodihydrofluorescein diacetate (DCFH-DA) probe, and an induction of cellular ROS was detected in response to CNP and DWCNT (50 μg/mL) treatment within 1 h in Ana-1/MHV-68 macrophages. ROS scavenging via *N*-acetylcysteine amide (NACA) at a nontoxic dose (Figure 4B,C) effectively abolished NP-triggered ROS formation (Figure 4D). However, NACA had no significant effect on MHV-68 reactivation caused by CNPs and DWCNTs as indicated by lytic viral gene expression (Figure 4E). Thus, exposure to NP-caused oxidative stress could not be causally linked with herpesvirus reactivation.

### NPs Activate MAPK Signaling without Proinflammatory Macrophage Activation

**Figure 5:**
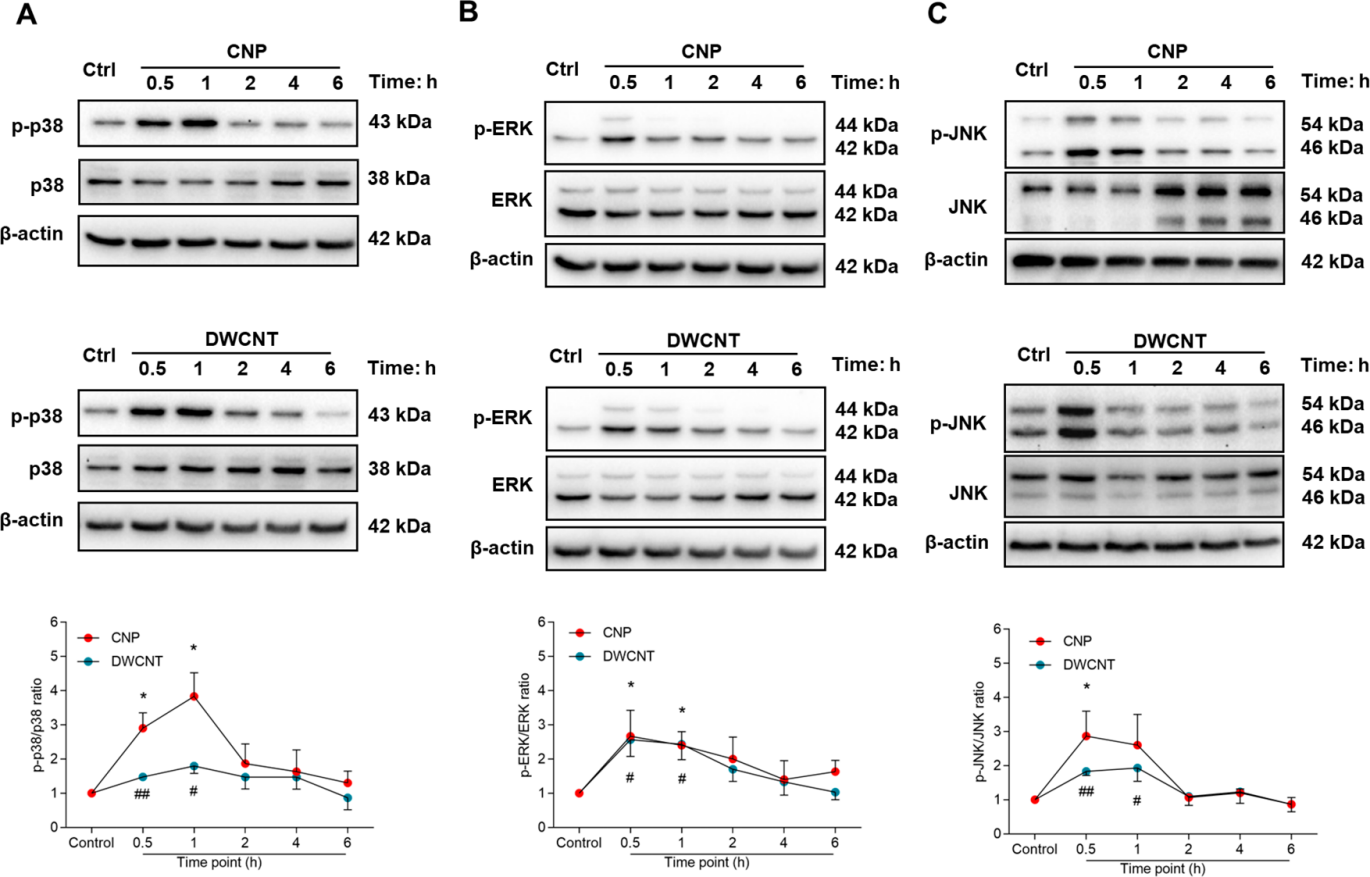
CNPs and DWCNTs activated the MAPK signaling pathway in Ana-1/MHV-68 cells. Ana-1/MHV-68 cells were exposed for 0.5 to 6 h to CNPs (50 μg/mL) or DWCNTs (50 μg/mL) or an equal amount of medium as sham control, and immunoblot analysis of MAPK activation was performed. Whole cell protein was assessed for p38, ERK, and JNK phosphorylation, with β-actin as loading control (*N* = 3–4). Representative blots show the highest levels 30 min and 1 h after CNP (upper panel) or DWCNT (lower panel) treatment for p38 (A), ERK (B), and JNK (C) phosphorylation. Quantification demonstrates induced phosphorylation levels for all three kinases between 30 and 60 min after particle treatment. Values are shown as mean ± SEM. Data were analyzed by Student’s *t* test; “*” indicates statistically significant difference between “CNP” and “control”, whereas “#” indicates statistically significant difference between “DWCNT” and “control”. *P < 0.05, ^#^*P* < 0.05, ^##^*P* < 0.01.

Mitogen-activated protein kinase (MAPK) signaling has been reported to be activated by certain particle–cell interactions as important stress response pathway in macrophages,^37,43^ and MAPK pathways are involved in herpesvirus infection and reactivation.^44,45^ Thus, we studied the kinase activation of p38, ERK, and JNK at early time points after CNP and DWCNT exposure in Ana-1/MHV-68 macrophages. Both NPs triggered p38, ERK, and JNK phosphorylation at early exposure times from 30 min to 1 h (Figure 5A–C).

**Figure 6:**
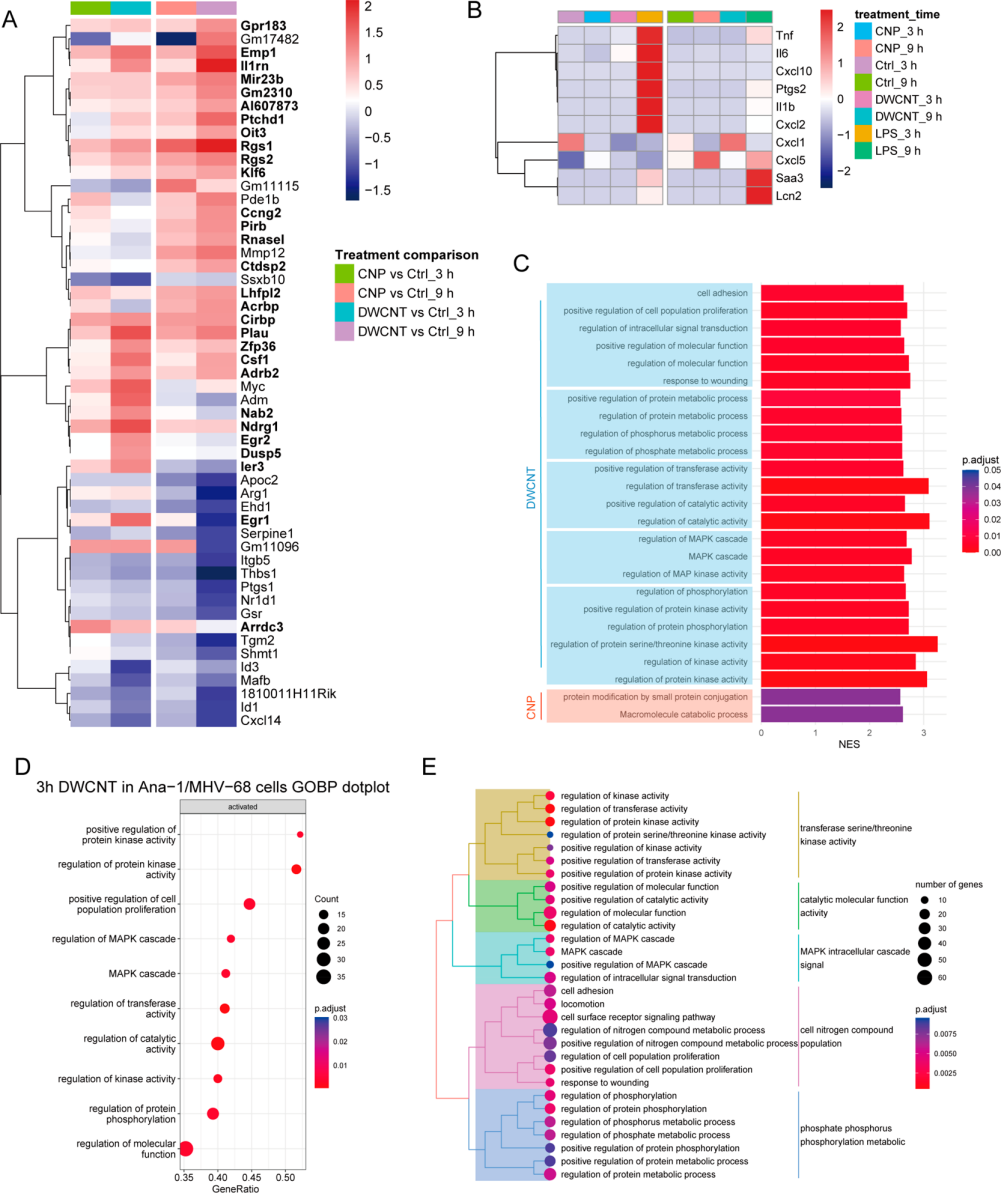
CNPs and DWCNTs do not cause a classical pro-inflammatory signature in exposed Ana-1 macrophages. (A) Microarray analysis was performed to detect transcriptomic changes caused by NP treatment. Ana-1/MHV-68 cells were exposed to CNPs (50 μg/mL), DWCNTs (50 μg/mL), LPS (1 μg/mL), or an equal amount of medium as control, and RNA was isolated after 3 and 9 h (*N* = 4). The transcriptional signature is represented by the heatmap arranged by hierarchical clustering to show genes with log2 fold change more than 1 or less than −1 compared to control (generated with R software, version 4.0.4; pheatmap package). Significantly expressed genes are highlighted in bold (*P* < 0.05, dabg). (B) A comparison of typical pro-inflammatory genes known for classical macrophage activation revealed the expected signature with early expression of *Tnf*, *Il1b*, *Cxcl2*, etc. and later *Lcn2*, *Saa3*, etc. for LPS- but not for CNP- or DWCNT-treated Ana-1/MHV-68 cells. (C) Gene Set Enrichment Analysis (GSEA) analysis on Gene Ontology Biological Process (GOBP) revealed distinct signaling activation for CNPs and DWCNTs (adjusted *p*-value < 0.05) after 3–9 h; the result is shown in bar plot; the length of the bar represents the normalized enrichment score (NES) and the color represents the adjusted *p*-value. Enriched terms with red bars are for CNPs, whereas those with blue bars are for DWCNTs. The analysis predicted the activation of “regulation of protein kinase activity”, “MAPK cascade”, and “regulation of protein phosphorylation” induced by DWNCTs. (D, E) Representative enrichment analysis results for DWCNT exposure after 3 h as well as enrichment term grouping are shown in dotplot and treeplot. Data was analyzed with R and Clusterprofiler package.

Since MAPK signaling is widely reported to promote proinflammatory cell activation states, and LPS (Figure 2D) was most effective in virus reactivation, we analyzed the inflammatory gene expression signature by microarray analysis after 3 and 9 h of CNP and DWCNT exposure, with LPS as a positive control. However, a classical proinflammatory macrophage signature could be detected only upon LPS but not CNP or DWCNT exposure (Figure 6A,B and Figure S4A,B). The lack of a NP-triggered pro-inflammatory macrophage state was further confirmed for classical pro-inflammatory macrophage markers by qPCR (Figure S4C). CNPs induced only 2 genes at least 2-fold and maximally 2.9-fold, and DWCNTs induced 19 genes at least 2-fold and maximally 4.3-fold within 9 h of treatment (significantly upregulated genes are labeled bold in the heatmap in Figure 6A). Gene Set Enrichment Analysis (GSEA) on the Gene Ontology Biological Process (GOBP) and further enrichment grouping revealed distinct enrichment for both NPs. Accordingly, CNP activated only two pathways, “protein modification by small protein conjugation” and “macromolecule catabolic process”, after 9 h, whereas DWCNTs activated “regulation of protein kinase activity”, “regulation of protein phosphorylation” and “regulation of MAPK cascade and catalytic activity” after 3–9 h exposure in Ana-1/MHV-68 cells, which fits to the activation of MAPK signaling described before (Figure 6C). Also, a deeper look into the top enrichments of GO terms as well as their grouping highlights kinase activity, protein phosphorylation, and MAPK signaling for DWCNT exposure at 3 h (Figure 6D,E). The lack of a transcriptional MAPK signaling signature for CNPs, in contrast to the described MAPK activation (Figure 5), might be explained by the weak transcriptional response and the low number of genes significantly regulated by CNPs.

### p38 Specific Inhibition Abolishes CNP- but not DWCNT-Induced Herpesvirus Reactivation

**Figure 7:**
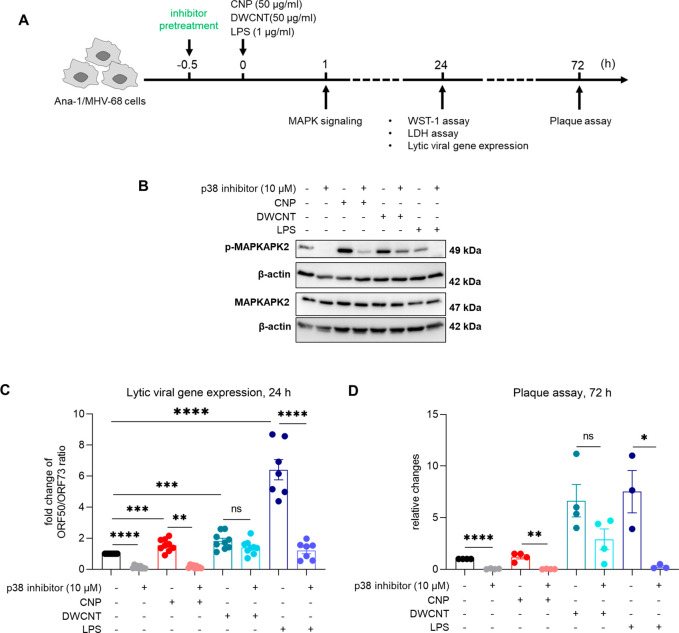
p38 inhibition efficiently inhibits the p38 MAPK signaling pathway and attenuates MHV-68 reactivation. The p38 selective inhibitor (SB203580) was used to examine the requirement of p38 MAPK signaling for NP induced MHV-68 reactivation. (A) Cells were pretreated 30 min before particle or LPS exposure with SB203580 and analyzed for MAPK signaling and virus reactivation at respective time points. (B) Western blot analysis proved the efficacy of p38 inhibition, not by reducing the CNP- or DWCNT-triggered p38 phosphorylation but by blocking the phosphorylation of its specific downstream kinase MAPKAPK2. (C) qPCR for ORF50 (specific for the lytic phase) and ORF73 (expressed during the lytic and latent phases) showed that p38 inhibition also blocked subsequent MHV-68 lytic gene expression induced by CNPs as well as LPS but not DWCNTs after 24 h. Fold changes of expression were calculated as ratio of ORF50 to ORF73 relative to controls (*N* = 7 for “LPS” and “LPS + p38 inhibitor”, *N* = 9 for the rest of the treatments). (D) Similarly, p38 inhibition also reduced the level of infectious MHV-68 titer induced by CNPs and LPS exposure after 72 h, but had no effect on DWCNT-induced MHV-68 reactivation (*N* = 3–4). All values in the figure are shown as mean ± SEM. Data were analyzed by Student’s *t* test; “*” indicates statistically significant difference versus “control”. *P < 0.05.

To investigate whether particle triggered MAPK signaling is required for MHV-68 reactivation, p38, ERK, and JNK MAPK specific inhibitors were applied 30 min prior to the NP treatment of Ana-1/MHV-68 cells (Figure 7A). At a concentration of 10 μM, which did not affect cytotoxicity or cell viability (Figure S5), pretreatment of Ana-1/MHV-68 cells with the p38 inhibitor, SB203580, effectively inhibited by CNP- and LPS-stimulated activity of the p38 specific downstream kinase MAPKAPK2 (Figure 7B). p38 inhibition, however, had only little effect on DWCNT-triggered MAPKAPK2 phosphorylation. Most importantly, p38 inhibition of infected macrophages also extinguished CNP-triggered lytic gene expression 24 h after stimulation, and because of the blockage of ORF50 induction, infectious virus production 72 h after stimulation was also inhibited (Figure 7C,D). A similar pattern was observed for LPS but not DWCNTs, in agreement with what was observed for MAPKAPK2 activation. ERK and JNK inhibitors were also tested, but no effect was found on reducing MHV-68 reactivation (data not shown).

We therefore conclude that CNP- but not DWCNT-triggered MHV-68 reactivation is dependent on the p38 MAPK signaling pathway and that targeting this pathway might offer a therapeutic target.

### p38 Inhibitor Pretreatment Attenuates CNP-Triggered Herpesvirus Reactivation In Vivo

**Figure 8:**
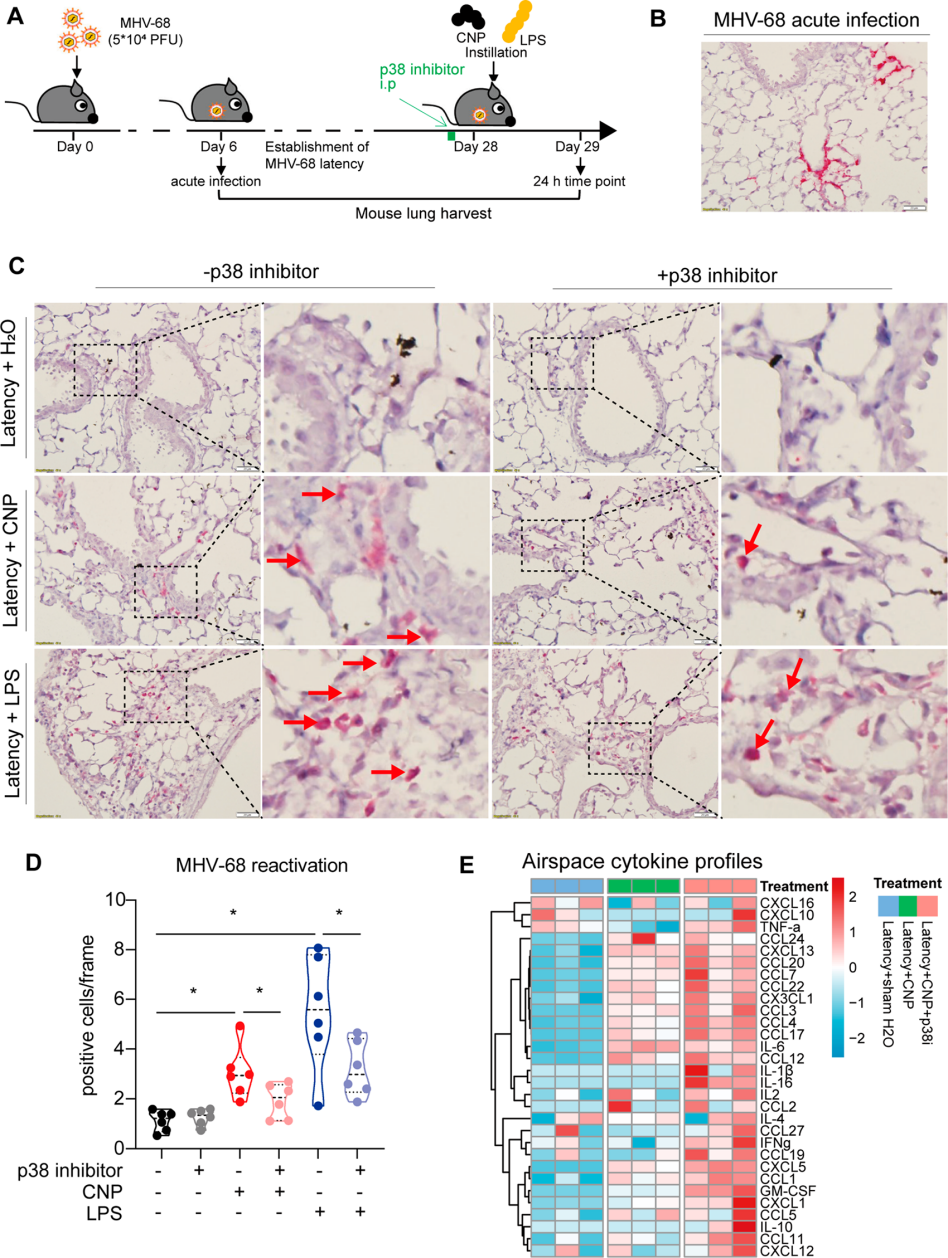
p38 inhibition attenuates MHV-68 reactivation in the lung of mice. (A) Schematic representation of animal experiment. C57BL/6 mice were intranasally infected with 5 × 10^4^ PFU of MHV-68 for 28 days to establish virus latency and subsequently instilled with either CNPs (50 μg) or LPS (positive control, 0.1 mg) or an equal amount of H_2_O as control. The p38 inhibitor (SB203580, 20 mg/kg) was intraperitoneally injected 1 h before particle instillation. Mouse lungs were harvested to investigate the effect of p38 inhibition on CNP-induced MHV-68 reactivation 24 h thereafter. (B) Immunohistochemistry (IHC) showed lytic virus protein expression during acute infection (6 days after infection) predominantly in airway epithelial cells. (C) IHC demonstrates that CNP and LPS exposure induced MHV-68 lytic protein expression, supporting MHV-68 reactivation (upper panel), and p38 inhibitor administration reduced reactivation related lytic protein expression (lower panel). IHC slides were stained with anti-MHV-68 serum, detected by Vulcan fast red and counterstained with hematoxylin. Scale bar: 20 μm. (D) Quantification of lytic virus protein expressing cells using the CAST method proved the attenuation of MHV-68 reactivation by p38 inhibition in the lungs of latently infected mice (*N* = 6). (E) BAL cytokine levels illustrated as heatmap (*N* = 3); *z*-score was computed as data normalization row-by-row. Data were analyzed by Student’s *t* test; “*” indicates statistically significant difference between displayed groups. *P < 0.05.

To investigate the applicability of the p38 inhibitor at the *in vivo* level (Figure 8A), C57BL/6 mice were intranasally (i.n.) infected with 5 × 10^4^ PFU of MHV-68 and, at day 28 postinfection when latency is established, intraperitoneally (i.p.) pretreated with SB203580 (20 mg/kg) or vehicle 1 h before CNP or LPS instillation. Twenty-four hours after particle exposure, the lungs were analyzed for virus reactivation. Immunohistochemistry (IHC) staining confirmed massive MHV-68 lytic virus protein expression predominantly localized to the epithelium 6 days after MHV-68 infection, i.e., during the acute infection phase (Figure 8B), and comparable to the whole mount analysis shown in Figure 2B. Compared to controls, 24 h after CNP exposure increased lytic protein expressing cells were detected in the lungs, indicating MHV-68 reactivation (Figure 8C,D). Quantification of MHV-68 positive cells demonstrates ∼3-fold increase of reactivating cells caused by CNPs and about 5.5-fold caused by LPS. Importantly, p38 inhibitor pretreatment attenuated CNP-triggered MHV-68 reactivation and reduced MHV-68 positive cell numbers by about 50%. In agreement with the *in vitro* study, a similar effect was found for LPS (Figure 8C,D). As it has been described for various nanoparticle toxicity studies,^46,47^ pulmonary CNP exposure caused an acute inflammatory response characterized by alveolar neutrophil accumulation (Figure S6B) and cytokine release into the airspace (Figure 8E). While several BAL cytokine levels related to granulocytes (IL6, GM-CSF, CXCL5, CCL3, and CCL20) or monocytes (CCL4 and CCL7) were increased 24 h after NP exposure, p38 inhibitor pretreatment did not dampen but rather enhanced their levels (Figure S6A), suggesting a boost of the local proinflammatory milieu in the airspace. As a consequence, MHV-68 reactivation seems not directly associated with the level of inflammation since p38 inhibition attenuated reactivation but not inflammation.

In summary, pulmonary CNP exposure triggered MHV-68 reactivation via a p38 MAPK dependent signaling pathway, with p38 inhibition providing a potential therapeutic target.

## Discussion

Usually sterile, uninfected animal or cell models are used to investigate the respiratory risk of nanoparticle exposure and nanosafety assessment in general, but our study highlights the requirement of latent infection for the susceptibility to develop carbon particle exposure related lung injury. In this context, we have previously shown that long-lasting, chronic responses to a single NP exposure can originate from the interplay of nanomaterial cycling between lung epithelial cells and macrophages, and this insight could be translated into a model to predict chronic lung inflammation.^48^ The predictive model worked for 10 metal oxide NPs (TiO_2_, SiO_2_, and ZnO) but not for carbonaceous CNPs or CNTs. Also other NPs such as TiO_2_ can trigger virus reactivation from latency^32^ suggesting that this effect is not material specific. Our current study suggests that latent virus infection generates a susceptibility to NP exposure which likely contributes to a long-lasting toxicity of inhaled nanoparticles, even after only two repeated NP exposures.

Environmental nanoparticle exposure and herpesvirus infections are worldwide and inevitable challenges to human health, and pulmonary exposure to nanoparticles can cause reactivation of a latent herpesvirus in mouse lungs and in human cell lines.^32^ Here, we demonstrate that repeated CNP or DWCNT exposure caused increased lung inflammation and alveolar injury with emphysema-like changes only in MHV-68 infected mouse lungs. Noteworthy, the level of airspace enlargement, developed within 1 week after the last NP exposure, corresponds to the level observed in our cigarette smoke induced COPD mouse models after 4 months of smoke exposure.^49,50^ Immunohistology revealed that pulmonary nanoparticle exposure induced virus reactivation, particularly in the lung parenchyma infiltrating macrophages. Our mechanistic studies demonstrated that CNP triggered MHV-68 reactivation via a p38 MAPK dependent signaling cascade, and preventive p38 inhibition significantly attenuated MHV-68 reactivation to 50% of the levels induced by CNPs. Even though DWCNTs also triggered p38 MAPK signaling, the contribution of this pathway was less relevant for virus reactivation.

Noteworthy, alveolar cell death and the loss of alveolar walls increased during the six day recovery period after the last NP dose, which points to a progressive pathology. In fact, this mode of action for emphysema-like lung injury caused by repeated particle exposure, dependent on reactivation of latent herpesvirus infection, might be an important factor contributing to COPD development in nonsmokers exposed to high levels of air pollution or other modes of particle burden. In this view, it has to be considered that usually healthy and not latently infected animal models are used in toxicological studies investigating health effects of nanoparticle exposure or air pollution, despite the fact that the majority of human adults carry various latent herpes virus infections.^26^ Accordingly, epidemiological studies reporting associations of long-term air pollution with increases in COPD and emphysema-like changes^16^ or other subclinical manifestations of chronic lung diseases^51^ may not be reproduced in mechanistic experimentation missing the contribution of virus reactivation from common latent infections. In contrast to our study which focused on the reactivation of an established latent herpesvirus infection by inhaled particles, Liu and colleagues described recently very elegantly how inhaled particles can synergistically effect influenza virus infection and biodistribution.^52^ Depending on their physicochemical properties, ambient air pollution particles may not only enhance influenza virus translocation into extrapulmonary organs but also facilitate virus internalization into host cells and promote budding and dispersal of progeny virions from the host cell plasma membrane.

Here, however, we were interested in understanding in which lung cell types the reactivation of latent virus occurs; however, by nature, the identification of the cell type that harbors the dormant virus during latency is rather difficult. γ-Herpesviruses have been reported to establish latency mainly in B cells, macrophages, and dendritic cells.^53^ In the present study, we found that LPS or nanoparticle exposure triggered reactivation in CD11b+ macrophage-like infiltrates but not CD11c+ tissue resident macrophages. Accordingly, obviously nanoparticle agglomerate laden alveolar macrophages did not express lytic virus proteins. The reactivating cells predominately detected in the interstitium were also positive for inflammatory monocyte or macrophage markers like AIF1 and GPNMB, besides CD11b, clearly distinguishing them from the alveolar macrophage lineage. Our immunohistochemical analysis revealed that during latency, about 1.5% of the lung cells showed MHV-68 lytic protein expression, which increased to about 2.8% after CNP treatment and to 4.4% after LPS treatment. In agreement, 1% of all CD11b+ cells were MHV-68 antigen-positive during latency, which increased to 2% after CNP exposure. A recent human study also showed that HHV-6 antigens detected in the parenchyma of IPF patient lungs localized to mononuclear inflammatory cells, potentially macrophages or lymphocytes.^54^ In our model, however, no lytic protein expression was detected in CD3+ T cells or B220+ B cells, even if CD8+ T cells accumulated in the lungs during establishment of latency. The importance of lung macrophages and their plastic interaction with the local environment has attracted increasing scientific interest, and we start to understand better how dysregulated macrophage behavior may contribute the development of CLDs.^55^ In this context, monocyte extravasation and accumulation resulting in tissue injury in COPD has been described by us and others,^50^ and γ-herpesvirus infected monocytes might show a mode of action not described so far.

Searching for the triggers causing virus reactivation *in vitro*, we confirmed that NP-exposure generated intracellular oxidative stress; however, scavenging particle-induced ROS with the antioxidant NACA failed to clearly attenuate NP-triggered MHV-68 reactivation. Ye et al. reported H_2_O_2_ triggered KSHV reactivation, which was abolished by NAC treatment.^39^ This contrast might suggest the relevance of other less NACA-quenchable and DCFH-sensitive reactive radical species for virus reactivation. The activation of MAPK signaling has already previously been reported to contribute to herpesvirus infection, and Ye et al. showed that H_2_O_2_ reactivates KSHV from latency via p38, ERK, and JNK MAPK signaling.^39^ However, in the present study, CNP exposure triggered herpesvirus reactivation dependent only on p38 MAPK signaling, whereas specific inhibition of ERK and JNK signaling even enhanced virus reactivation. ERK inhibition also induced proapoptotic pathways, detected by increased caspase-3 cleavage (data not shown), which could explain the observed virus reactivation due to cell stress. For ethical reasons, we have tested only p38 inhibition in the mouse model, and here, we confirmed that systemic pretreatment with SB203580 preventively attenuated MHV-68 reactivation induced by pulmonary CNP exposure. Thus, p38 might serve as potential therapeutic target in emphysematous CLDs related to ambient particle exposure, and p38 inhibition is already a drug target for chronic inflammatory lung diseases such as COPD.^56,57^ Even if the results of clinical trials evaluating p38 MAPK inhibitors as drugs for COPD therapy are currently controversial,^58^ this could be different for COPD observed in never-smokers where exposure and mechanisms of disease development might differ as well.

Recurrent lung epithelial injury is widely discussed to contribute to CLD pathogenesis.^59^ In the current animal study, we have focused on the mechanism of NP- exposure-triggered γ-herpesvirus reactivation using only one or two particle exposures as a second hit scenario. In a real-world scenario, however, for most people living at respective urban sites or suffering from occupational exposure, a plethora of air pollution episodes and recurrences of exposures to high particle number levels are occurring. According to our finding, reoccurring exposure will cause repeated virus reactivation with corresponding local and systemic immunomodulation, which may contribute to persistent lung injury and damage. Our future animal experiments will mimic this situation by several repetitive hit scenarios, i.e., up to five repeated particle dosing of latently infected mice, to study potentially escalating consequences.

## Conclusions

In summary, we demonstrated that pulmonary repeated carbon nanoparticle (CNP) or DWCNT exposure caused elevated lung inflammation, lung injury, and notably progressive alveolar cell death with lung emphysema in an animal model of murine γ-herpesvirus infection. We further show that soot-like nanoparticles trigger γ-herpesvirus reactivation in macrophage infiltrates of latently infected lungs via a p38 MAPK dependent and pharmacologically druggable pathway. Therapeutic p38 inhibition might thus not only prevent virus reactivation but also alleviate ambient or occupational particle exposure related lung pathologies or emphysematous COPD like diseases. And future nanosafety studies and approaches to predict the risk of NP exposure should consider the susceptibility conferred by an omnipresent latent viral infection.

## Methods

Additional method details can be found in the Supporting Information.

### Materials and Reagents

CNPs (Printex90, Degussa, Frankfurt, Germany) and DWCNTs (Nanocyl, SA, Belgium) were used in this study. For *in vitro* experiments, 3 mg of CNPs or DWCNTs was weighed and dispersed with 3 mL of ultrapure H_2_O in a sterile glass tube to get a 1 mg/mL stock solution. For *in vivo* experiments, 3 mg of CNPs and DWCNTs was dispersed in 3 mL of ultrapure H_2_O, and the DWCNT dispersion was supplemented with 1% Pluronic F-127 (Sigma-Aldrich, Germany) as described earlier.^32^ Pluronic F-127 is an FDA approved surfactant to facilitate dispersion quality. CNP and DWCNT dispersions were sonicated in an ice-cold water bath for 5 min followed by 30 s Bioruptor probe sonication for CNP dispersion and 50 s Bioruptor probe sonication for DWCNT dispersion (Diagenode, Liege, Belgium) at 30% power and continuous mode, followed by 5 min sonication in an ice-cold water bath. The average size (*z*-average) and size distribution (represented by the polydispersity index = PDI) of NPs dispersed in water, medium, or 1% Pluronic F-127 in water was determined by photon correlation spectroscopy using a Dynamic Laser Scatter (DLS) Zetasizer Nano ZS (Malvern Instruments Ltd., Malvern, UK) as described in the literature.^60^ The absence of endotoxin from the particle preparations by the LIMULUS assay and the usage of a LPS sensitive macrophage cell line was approved. For *in vivo* experiments, mice were instilled with 50 μg of NPs. For *in vitro* experiments, 50 μL of stock NP solution was diluted in 950 μL of cell culture medium to reach a 50 μg/mL working concentration. Detailed characterizations and dispersion quality of both NPs are shown in Tables S1 and S2.

The Ana-1 cell line,^61,62^ Ana-1 cells latently infected with MHV-68 (Ana-1/MHV-68 cells), and BHK-21 cells (ATCC CCL-10) were used in the study.

### Determination of Gene Expression by qPCR

Whole cell RNA was isolated with the NucleoSpin RNA Plus kit (MACHEREY-NAGEL, Duren, Germany) following the instructions of the manufacturer. Subsequently, RNA was reverse-transcribed using a superscript kit (Invitrogen, Waltham, MA, USA). Then, cDNA was used to analyze the target gene expression by real time quantitative PCR using SYBR Green PCR master mix (Thermo fisher, Waltham, MA, USA).

### Determination of Virus Titer by Plaque Assay

Virus production into supernatant was quantified by standard plaque assay on BHK-21 cells as previously described.^32^

### Transcriptomic Study by Microarray Analysis

Total RNA from Ana-1/MHV-68 cells exposed to CNPs, DWCNTs, LPS, or control after 3 and 9 h was used for microarray analysis to investigate the transcriptomic changes. Detailed information can be found in Supporting Information.

### In Vivo Experiments

C57BL/6 mice were purchased from Charles River Laboratories and housed in individually ventilated cages (IVCs) during the MHV-68 infection period. Mice were anesthetized with midazolam/medetomidine/fentanyl (MMF) and infected i.n. with 5 × 10^4^ PFU of MHV-68. After 28 days, mice were instilled with 50 μg of CNPs, 0.1 μg of LPS, or an equal amount sterile H_2_O as sham control per mouse as described previously.^32^ For repeated CNP exposure, instillation was performed for a second time after 55 days. For the p38 inhibitor experiment, 30 mg/kg p38 inhibitor (SB203580; Merck, Darmstadt, Germany) was administered by intraperitoneal injection (ip) in each mouse 1 h before instillation. After 24 h and 6 days, lung tissue was either harvested for protein, RNA, and DNA isolation or fixed with 4% paraformaldehyde (PFA) solution for histology analysis. Bronchoalveolar lavage (BAL) fluid and cells were also collected for the assessment of inflammatory cell and cytokine profiles. All animal experiments were in compliance with protocols approved by the local Animal Care and Use Committee (District Government of Upper Bavaria; permit number: 55.2-2532.Vet_02-15-67).

### Light-Sheet Fluorescence Microscopy (LSFM)

Mouse lung lobes were stained with MHV-68 antiserum or were used to perform optical clearing according to the 3DISCO protocol.^63^

### Graphics

The graphical abstract was generated by Figdraw (www.figdraw.com).

### Statistical Analysis

All values are shown as mean ± SEM. Comparisons between two groups with normal distribution were performed with Student’s *t* test or by Mann–Whitney test when they were non-normally distributed. Multiple groups were compared with one-way ANOVA followed by Tukey’s multiple comparisons test using the GraphPad Prism software v9.0 (GraphPad Software, Inc., San Diego, CA, USA). “*” was used to show significant difference: *P < 0.05, **P < 0.01, ***P < 0.001, ****P < 0.0001. Statistical analysis of the transcriptomic study is described in the section “Transcriptomic Study by Microarray Analysis”.

## Supporting Information Available

Detailed methods; Table S1, characterization of NPs in the study; Table S2, average size and size distribution; Table S3, primer pairs used in this study; Figure S1, repeated NP exposure reactivates MHV-68 and increases interstitial inflammation, alveolar air space, and epithelial–endothelial barrier disruption; Figure S2, MHV-68 reactivating macrophages show no nanoparticle agglomerates burden; Figure S3, alveolar macrophages but not recruited macrophages are the main components in bronchoalveolar lavage (BAL) cells and hardly showed MHV-68 reactivation; Figure S4, a strong pro-inflammatory response was induced by LPS exposure; Figure S5, p38 inhibition showed no further toxicity to Ana-1/MHV-68 cells; Figure S6, CNP exposure caused airspace inflammation and is not attenuated by p38 inhibition; abbreviations; references (PDF)

## References

[ref1] Brunelli A.; Calgaro L.; Semenzin E.; Cazzagon V.; Giubilato E.; Marcomini A.; Badetti E. Leaching of Nanoparticles from Nano-Enabled Products for the Protection of Cultural Heritage Surfaces: A Review. Environmental Sciences Europe 2021, 33 (1), 48 10.1186/s12302-021-00493-z.

[ref2] WHO Air Pollution. https://www.who.int/health-topics/air-pollution#tab=tab_1. Last accessed date: February 2, 2023.

[ref3] Andersen Z. J.; Hvidberg M.; Jensen S. S.; Ketzel M.; Loft S.; Sørensen M.; Tjønneland A.; Overvad K.; Raaschou-Nielsen O. Chronic Obstructive Pulmonary Disease and Long-Term Exposure to Traffic-Related Air Pollution: A Cohort Study. Am. J. Respir Crit Care Med. 2011, 183 (4), 455–61. 10.1164/rccm.201006-0937OC. 20870755

[ref4] Shin S.; Bai L.; Burnett R. T.; Kwong J. C.; Hystad P.; van Donkelaar A.; Lavigne E.; Weichenthal S.; Copes R.; Martin R. V.; Kopp A.; Chen H. Air Pollution as a Risk Factor for Incident Chronic Obstructive Pulmonary Disease and Asthma. A 15-Year Population-Based Cohort Study. Am. J. Respir Crit Care Med. 2021, 203 (9), 1138–1148. 10.1164/rccm.201909-1744OC. 33147059

[ref5] Wang L.; Xie J.; Hu Y.; Tian Y. Air Pollution and Risk of Chronic Obstructed Pulmonary Disease: The Modifying Effect of Genetic Susceptibility and Lifestyle. EBioMedicine 2022, 79, 103994 10.1016/j.ebiom.2022.103994. 35417845 PMC9018147

[ref6] Mills N. L.; Donaldson K.; Hadoke P. W.; Boon N. A.; MacNee W.; Cassee F. R.; Sandström T.; Blomberg A.; Newby D. E. Adverse Cardiovascular Effects of Air Pollution. Nat. Clin Pract Cardiovasc Med. 2009, 6 (1), 36–44. 10.1038/ncpcardio1399. 19029991

[ref7] André E.; Stoeger T.; Takenaka S.; Bahnweg M.; Ritter B.; Karg E.; Lentner B.; Reinhard C.; Schulz H.; Wjst M. Inhalation of Ultrafine Carbon Particles Triggers Biphasic Pro-Inflammatory Response in the Mouse Lung. Eur. Respir. J. 2006, 28 (2), 275–85. 10.1183/09031936.06.00071205. 16641123

[ref8] Ganguly K.; Ettehadieh D.; Upadhyay S.; Takenaka S.; Adler T.; Karg E.; Krombach F.; Kreyling W. G.; Schulz H.; Schmid O.; Stoeger T. Early Pulmonary Response Is Critical for Extra-Pulmonary Carbon Nanoparticle Mediated Effects: Comparison of Inhalation Versus Intra-Arterial Infusion Exposures in Mice. Part Fibre Toxicol 2017, 14 (1), 19 10.1186/s12989-017-0200-x. 28637465 PMC5480131

[ref9] Shvedova A. A.; Kisin E. R.; Mercer R.; Murray A. R.; Johnson V. J.; Potapovich A. I.; Tyurina Y. Y.; Gorelik O.; Arepalli S.; Schwegler-Berry D.; Hubbs A. F.; Antonini J.; Evans D. E.; Ku B. K.; Ramsey D.; Maynard A.; Kagan V. E.; Castranova V.; Baron P. Unusual Inflammatory and Fibrogenic Pulmonary Responses to Single-Walled Carbon Nanotubes in Mice. Am. J. Physiol Lung Cell Mol. Physiol 2005, 289 (5), L698–708. 10.1152/ajplung.00084.2005. 15951334

[ref10] Vietti G.; Lison D.; van den Brule S. Mechanisms of Lung Fibrosis Induced by Carbon Nanotubes: Towards an Adverse Outcome Pathway (Aop). Part Fibre Toxicol 2015, 13, 11 10.1186/s12989-016-0123-y. PMC4772332 26926090

[ref11] Rydman E. M.; Ilves M.; Koivisto A. J.; Kinaret P. A.; Fortino V.; Savinko T. S.; Lehto M. T.; Pulkkinen V.; Vippola M.; Hämeri K. J.; Matikainen S.; Wolff H.; Savolainen K. M.; Greco D.; Alenius H. Inhalation of Rod-Like Carbon Nanotubes Causes Unconventional Allergic Airway Inflammation. Part Fibre Toxicol 2014, 11, 48 10.1186/s12989-014-0048-2. 25318534 PMC4215016

[ref12] Bonner J. C. Nanoparticles as a Potential Cause of Pleural and Interstitial Lung Disease. Proc. Am. Thorac Soc. 2010, 7 (2), 138–41. 10.1513/pats.200907-061RM. 20427587 PMC3266021

[ref13] Wang P.; Nie X.; Wang Y.; Li Y.; Ge C.; Zhang L.; Wang L.; Bai R.; Chen Z.; Zhao Y.; Chen C. Multiwall Carbon Nanotubes Mediate Macrophage Activation and Promote Pulmonary Fibrosis through Tgf-B/Smad Signaling Pathway. Small 2013, 9 (22), 3799–811. 10.1002/smll.201300607. 23650105

[ref14] Kasai T.; Umeda Y.; Ohnishi M.; Mine T.; Kondo H.; Takeuchi T.; Matsumoto M.; Fukushima S. Lung Carcinogenicity of Inhaled Multi-Walled Carbon Nanotube in Rats. Part Fibre Toxicol 2015, 13 (1), 53 10.1186/s12989-016-0164-2. PMC5064785 27737701

[ref15] You R.; Lu W.; Shan M.; Berlin J. M.; Samuel E. L.; Marcano D. C.; Sun Z.; Sikkema W. K.; Yuan X.; Song L.; Hendrix A. Y.; Tour J. M.; Corry D. B.; Kheradmand F. Nanoparticulate Carbon Black in Cigarette Smoke Induces DNA Cleavage and Th17-Mediated Emphysema. Elife 2015, 4, e09623 10.7554/eLife.09623. 26437452 PMC4612775

[ref16] Wang M.; Aaron C. P.; Madrigano J.; Hoffman E. A.; Angelini E.; Yang J.; Laine A.; Vetterli T. M.; Kinney P. L.; Sampson P. D.; Sheppard L. E.; Szpiro A. A.; Adar S. D.; Kirwa K.; Smith B.; Lederer D. J.; Diez-Roux A. V.; Vedal S.; Kaufman J. D.; Barr R. G. Association between Long-Term Exposure to Ambient Air Pollution and Change in Quantitatively Assessed Emphysema and Lung Function. Jama 2019, 322 (6), 546–556. 10.1001/jama.2019.10255. 31408135 PMC6692674

[ref17] Britto C. J.; Brady V.; Lee S.; Dela Cruz C. S. Respiratory Viral Infections in Chronic Lung Diseases. Clin Chest Med. 2017, 38 (1), 87–96. 10.1016/j.ccm.2016.11.014. 28159164 PMC5679206

[ref18] Tan K. S.; Lim R. L.; Liu J.; Ong H. H.; Tan V. J.; Lim H. F.; Chung K. F.; Adcock I. M.; Chow V. T.; Wang Y. Respiratory Viral Infections in Exacerbation of Chronic Airway Inflammatory Diseases: Novel Mechanisms and Insights from the Upper Airway Epithelium. Front Cell Dev Biol. 2020, 8, 99 10.3389/fcell.2020.00099. 32161756 PMC7052386

[ref19] Linden D.; Guo-Parke H.; Coyle P. V.; Fairley D.; McAuley D. F.; Taggart C. C.; Kidney J. Respiratory Viral Infection: A Potential “Missing Link” in the Pathogenesis of COPD. Eur. Respir Rev. 2019, 28 (151), 180063 10.1183/16000617.0063-2018. 30872396 PMC9488189

[ref20] White D. W.; Suzanne Beard R.; Barton E. S. Immune Modulation During Latent Herpesvirus Infection. Immunol Rev. 2012, 245 (1), 189–208. 10.1111/j.1600-065X.2011.01074.x. 22168421 PMC3243940

[ref21] Adler B.; Sattler C.; Adler H. Herpesviruses and Their Host Cells: A Successful Liaison. Trends Microbiol 2017, 25 (3), 229–241. 10.1016/j.tim.2016.11.009. 27956142

[ref22] Stoeger T.; Adler H. ″Novel″ Triggers of Herpesvirus Reactivation and Their Potential Health Relevance. Front Microbiol 2019, 9, 3207 10.3389/fmicb.2018.03207. 30666238 PMC6330347

[ref23] Aligo J.; Walker M.; Bugelski P.; Weinstock D. Is Murine Gammaherpesvirus-68 (Mhv-68) a Suitable Immunotoxicological Model for Examining Immunomodulatory Drug-Associated Viral Recrudescence?. J. Immunotoxicol 2015, 12 (1), 1–15. 10.3109/1547691X.2014.882996. 24512328

[ref24] Hair J. R.; Lyons P. A.; Smith K. G. C.; Efstathiou S. Control of Rta Expression Critically Determines Transcription of Viral and Cellular Genes Following Gammaherpesvirus Infection. J. Gen Virol 2007, 88, 1689–1697. 10.1099/vir.0.82548-0. 17485528 PMC2884955

[ref25] Pavlova I.; Lin C. Y.; Speck S. H. Murine Gammaherpesvirus 68 Rta-Dependent Activation of the Gene 57 Promoter. Virology 2005, 333 (1), 169–79. 10.1016/j.virol.2004.12.021. 15708602

[ref26] Kuri A.; Jacobs B. M.; Vickaryous N.; Pakpoor J.; Middeldorp J.; Giovannoni G.; Dobson R. Epidemiology of Epstein-Barr Virus Infection and Infectious Mononucleosis in the United Kingdom. BMC Public Health 2020, 20 (1), 912 10.1186/s12889-020-09049-x. 32532296 PMC7291753

[ref27] McManus T. E.; Marley A. M.; Baxter N.; Christie S. N.; Elborn J. S.; O’Neill H. J.; Coyle P. V.; Kidney J. C. High Levels of Epstein-Barr Virus in Copd. Eur. Respir. J. 2008, 31 (6), 1221–6. 10.1183/09031936.00107507. 18287127

[ref28] Chen C. L.; Huang Y.; Martinez-Garcia M. A.; Yuan J. J.; Li H. M.; de la Rosa-Carrillo D.; Han X. R.; Chen R. C.; Guan W. J.; Zhong N. S. The Role of Epstein-Barr Virus in Adults with Bronchiectasis: A Prospective Cohort Study. Open Forum Infect Dis 2020, 7 (8), ofaa235 10.1093/ofid/ofaa235. 32766379 PMC7397835

[ref29] Mora A. L.; Woods C. R.; Garcia A.; Xu J.; Rojas M.; Speck S. H.; Roman J.; Brigham K. L.; Stecenko A. A. Lung Infection with Gamma-Herpesvirus Induces Progressive Pulmonary Fibrosis in Th2-Biased Mice. Am. J. Physiol Lung Cell Mol. Physiol 2005, 289 (5), L711–21. 10.1152/ajplung.00007.2005. 15734789

[ref30] Mora A. L.; Torres-González E.; Rojas M.; Xu J.; Ritzenthaler J.; Speck S. H.; Roman J.; Brigham K.; Stecenko A. Control of Virus Reactivation Arrests Pulmonary Herpesvirus-Induced Fibrosis in Ifn-Gamma Receptor-Deficient Mice. Am. J. Respir Crit Care Med. 2007, 175 (11), 1139–50. 10.1164/rccm.200610-1426OC. 17363768 PMC1899276

[ref31] Sheng G.; Chen P.; Wei Y.; Yue H.; Chu J.; Zhao J.; Wang Y.; Zhang W.; Zhang H. L. Viral Infection Increases the Risk of Idiopathic Pulmonary Fibrosis: A Meta-Analysis. Chest 2020, 157 (5), 1175–1187. 10.1016/j.chest.2019.10.032. 31730835 PMC7214095

[ref32] Sattler C.; Moritz F.; Chen S.; Steer B.; Kutschke D.; Irmler M.; Beckers J.; Eickelberg O.; Schmitt-Kopplin P.; Adler H.; Stoeger T. Nanoparticle Exposure Reactivates Latent Herpesvirus and Restores a Signature of Acute Infection. Part Fibre Toxicol 2017, 14 (1), 2 10.1186/s12989-016-0181-1. 28069010 PMC5223553

[ref33] Pan H.; Xie J.; Ye F.; Gao S. J. Modulation of Kaposi’s Sarcoma-Associated Herpesvirus Infection and Replication by Mek/Erk, Jnk, and P38 Multiple Mitogen-Activated Protein Kinase Pathways During Primary Infection. J. Virol 2006, 80 (11), 5371–82. 10.1128/JVI.02299-05. 16699017 PMC1472170

[ref34] Stahl J. A.; Chavan S. S.; Sifford J. M.; MacLeod V.; Voth D. E.; Edmondson R. D.; Forrest J. C. Phosphoproteomic Analyses Reveal Signaling Pathways That Facilitate Lytic Gammaherpesvirus Replication. PLoS Pathog 2013, 9 (9), e1003583 10.1371/journal.ppat.1003583. 24068923 PMC3777873

[ref35] Stahl J. A.; Paden C. R.; Chavan S. S.; MacLeod V.; Edmondson R. D.; Speck S. H.; Forrest J. C. Amplification of Jnk Signaling Is Necessary to Complete the Murine Gammaherpesvirus 68 Lytic Replication Cycle. J. Virol 2012, 86 (24), 13253–62. 10.1128/JVI.01432-12. 23015701 PMC3503053

[ref36] Xie J.; Ajibade A. O.; Ye F.; Kuhne K.; Gao S. J. Reactivation of Kaposi’s Sarcoma-Associated Herpesvirus from Latency Requires Mek/Erk, Jnk and P38 Multiple Mitogen-Activated Protein Kinase Pathways. Virology 2008, 371 (1), 139–54. 10.1016/j.virol.2007.09.040. 17964626 PMC2239004

[ref37] Fritsch-Decker S.; Marquardt C.; Stoeger T.; Diabaté S.; Weiss C. Revisiting the Stress Paradigm for Silica Nanoparticles: Decoupling of the Anti-Oxidative Defense, Pro-Inflammatory Response and Cytotoxicity. Arch. Toxicol. 2018, 92 (7), 2163–2174. 10.1007/s00204-018-2223-y. 29799070

[ref38] Stoeger T.; Takenaka S.; Frankenberger B.; Ritter B.; Karg E.; Maier K.; Schulz H.; Schmid O. Deducing in Vivo Toxicity of Combustion-Derived Nanoparticles from a Cell-Free Oxidative Potency Assay and Metabolic Activation of Organic Compounds. Environ. Health Perspect 2009, 117 (1), 54–60. 10.1289/ehp.11370. 19165387 PMC2627865

[ref39] Ye F.; Zhou F.; Bedolla R. G.; Jones T.; Lei X.; Kang T.; Guadalupe M.; Gao S. J. Reactive Oxygen Species Hydrogen Peroxide Mediates Kaposi’s Sarcoma-Associated Herpesvirus Reactivation from Latency. PLoS Pathog 2011, 7 (5), e1002054 10.1371/journal.ppat.1002054. 21625536 PMC3098240

[ref40] Misharin A. V.; Morales-Nebreda L.; Mutlu G. M.; Budinger G. R.; Perlman H. Flow Cytometric Analysis of Macrophages and Dendritic Cell Subsets in the Mouse Lung. Am. J. Respir. Cell Mol. Biol. 2013, 49 (4), 503–10. 10.1165/rcmb.2013-0086MA. 23672262 PMC3824047

[ref41] Nel A.; Xia T.; Mädler L.; Li N. Toxic Potential of Materials at the Nanolevel. Science 2006, 311 (5761), 622–7. 10.1126/science.1114397. 16456071

[ref42] Li X.; Feng J.; Sun R. Oxidative Stress Induces Reactivation of Kaposi’s Sarcoma-Associated Herpesvirus and Death of Primary Effusion Lymphoma Cells. J. Virol 2011, 85 (2), 715–24. 10.1128/JVI.01742-10. 21068240 PMC3020037

[ref43] Qin Y.; Zhou Z. W.; Pan S. T.; He Z. X.; Zhang X.; Qiu J. X.; Duan W.; Yang T.; Zhou S. F. Graphene Quantum Dots Induce Apoptosis, Autophagy, and Inflammatory Response Via P38 Mitogen-Activated Protein Kinase and Nuclear Factor-Kb Mediated Signaling Pathways in Activated Thp-1 Macrophages. Toxicology 2015, 327, 62–76. 10.1016/j.tox.2014.10.011. 25446327

[ref44] Cohen A.; Brodie C.; Sarid R. An Essential Role of Erk Signalling in Tpa-Induced Reactivation of Kaposi’s Sarcoma-Associated Herpesvirus. J. Gen Virol 2006, 87 (4), 795–802. 10.1099/vir.0.81619-0. 16528027

[ref45] Qin D.; Feng N.; Fan W.; Ma X.; Yan Q.; Lv Z.; Zeng Y.; Zhu J.; Lu C. Activation of Pi3k/Akt and Erk Mapk Signal Pathways Is Required for the Induction of Lytic Cycle Replication of Kaposi’s Sarcoma-Associated Herpesvirus by Herpes Simplex Virus Type 1. BMC Microbiol 2011, 11, 240 10.1186/1471-2180-11-240. 22032493 PMC3215226

[ref46] Chen S.; Yin R.; Mutze K.; Yu Y.; Takenaka S.; Königshoff M.; Stoeger T. No Involvement of Alveolar Macrophages in the Initiation of Carbon Nanoparticle Induced Acute Lung Inflammation in Mice. Part Fibre Toxicol 2015, 13 (1), 33 10.1186/s12989-016-0144-6. PMC4915176 27328634

[ref47] Ma J.; Liu R.; Wang X.; Liu Q.; Chen Y.; Valle R. P.; Zuo Y. Y.; Xia T.; Liu S. Crucial Role of Lateral Size for Graphene Oxide in Activating Macrophages and Stimulating Pro-Inflammatory Responses in Cells and Animals. ACS Nano 2015, 9 (10), 10498–515. 10.1021/acsnano.5b04751. 26389709 PMC5522963

[ref48] Kokot H.; Kokot B.; Sebastijanović A.; Voss C.; Podlipec R.; Zawilska P.; Berthing T.; Ballester-López C.; Danielsen P. H.; Contini C.; Ivanov M.; Krišelj A.; Čotar P.; Zhou Q.; Ponti J.; Zhernovkov V.; Schneemilch M.; Doumandji Z.; Pušnik M.; Umek P.; Pajk S.; Joubert O.; Schmid O.; Urbančič I.; Irmler M.; Beckers J.; Lobaskin V.; Halappanavar S.; Quirke N.; Lyubartsev A. P.; Vogel U.; Koklič T.; Stoeger T.; Štrancar J. Prediction of Chronic Inflammation for Inhaled Particles: The Impact of Material Cycling and Quarantining in the Lung Epithelium. Adv. Mater. 2020, 32 (47), e2003913 10.1002/adma.202003913. 33073368

[ref49] Conlon T. M.; John-Schuster G.; Heide D.; Pfister D.; Lehmann M.; Hu Y.; Ertüz Z.; Lopez M. A.; Ansari M.; Strunz M.; Mayr C.; Angelidis I.; Ciminieri C.; Costa R.; Kohlhepp M. S.; Guillot A.; Günes G.; Jeridi A.; Funk M. C.; Beroshvili G.; Prokosch S.; Hetzer J.; Verleden S. E.; Alsafadi H.; Lindner M.; Burgstaller G.; Becker L.; Irmler M.; Dudek M.; Janzen J.; Goffin E.; Gosens R.; Knolle P.; Pirotte B.; Stoeger T.; Beckers J.; Wagner D.; Singh I.; Theis F. J.; de Angelis M. H.; O’Connor T.; Tacke F.; Boutros M.; Dejardin E.; Eickelberg O.; Schiller H. B.; Königshoff M.; Heikenwalder M.; Yildirim A. Inhibition of Ltβr Signalling Activates Wnt-Induced Regeneration in Lung. Nature 2020, 588 (7836), 151–156. 10.1038/s41586-020-2882-8. 33149305 PMC7718297

[ref50] Günes Günsel G.; Conlon T. M.; Jeridi A.; Kim R.; Ertüz Z.; Lang N. J.; Ansari M.; Novikova M.; Jiang D.; Strunz M.; Gaianova M.; Hollauer C.; Gabriel C.; Angelidis I.; Doll S.; Pestoni J. C.; Edelmann S. L.; Kohlhepp M. S.; Guillot A.; Bassler K.; Van Eeckhoutte H. P.; Kayalar Ö.; Konyalilar N.; Kanashova T.; Rodius S.; Ballester-López C.; Genes Robles C. M.; Smirnova N.; Rehberg M.; Agarwal C.; Krikki I.; Piavaux B.; Verleden S. E.; Vanaudenaerde B.; Königshoff M.; Dittmar G.; Bracke K. R.; Schultze J. L.; Watz H.; Eickelberg O.; Stoeger T.; Burgstaller G.; Tacke F.; Heissmeyer V.; Rinkevich Y.; Bayram H.; Schiller H. B.; Conrad M.; Schneider R.; Yildirim A. The Arginine Methyltransferase Prmt7 Promotes Extravasation of Monocytes Resulting in Tissue Injury in Copd. Nat. Commun. 2022, 13 (1), 1303 10.1038/s41467-022-28809-4. 35288557 PMC8921220

[ref51] Sack C.; Vedal S.; Sheppard L.; Raghu G.; Barr R. G.; Podolanczuk A.; Doney B.; Hoffman E. A.; Gassett A.; Hinckley-Stukovsky K.; Williams K.; Kawut S.; Lederer D. J.; Kaufman J. D. Air Pollution and Subclinical Interstitial Lung Disease: The Multi-Ethnic Study of Atherosclerosis (Mesa) Air-Lung Study. Eur. Respir. J. 2017, 50 (6), 1700559 10.1183/13993003.00559-2017. 29217611 PMC5726423

[ref52] Dong Z.; Ma J.; Qiu J.; Ren Q.; Shan Q.; Duan X.; Li G.; Zuo Y. Y.; Qi Y.; Liu Y.; Liu G.; Lynch I.; Fang M.; Liu S. Airborne Fine Particles Drive H1N1 Viruses Deep into the Lower Respiratory Tract and Distant Organs. Sci. Adv. 2023, 9 (23), eadf2165 10.1126/sciadv.adf2165. 37294770 PMC10256160

[ref53] Flaño E.; Husain S. M.; Sample J. T.; Woodland D. L.; Blackman M. A. Latent Murine Gamma-Herpesvirus Infection Is Established in Activated B Cells, Dendritic Cells, and Macrophages. J. Immunol 2000, 165 (2), 1074–81. 10.4049/jimmunol.165.2.1074. 10878386

[ref54] Pulkkinen V.; Salmenkivi K.; Kinnula V. L.; Sutinen E.; Halme M.; Hodgson U.; Lehto J.; Jääskeläinen A.; Piiparinen H.; Kere J.; Lautenschlager I.; Lappalainen M.; Myllärniemi M. A Novel Screening Method Detects Herpesviral DNA in the Idiopathic Pulmonary Fibrosis Lung. Ann. Med. 2012, 44 (2), 178–86. 10.3109/07853890.2010.532151. 21254895

[ref55] Bain C. C.; MacDonald A. S. The Impact of the Lung Environment on Macrophage Development, Activation and Function: Diversity in the Face of Adversity. Mucosal Immunol 2022, 15 (2), 223–234. 10.1038/s41385-021-00480-w. 35017701 PMC8749355

[ref56] Yu H.; Su X.; Lei T.; Zhang L.; Feng Z.; Zhang C.; Zhang M.; Wang Y.; Chen X.; Liu J. Safety and Efficacy of P38 Mitogen-Activated Protein Kinase Inhibitors (Mapkis) in Copd. Front Pharmacol 2022, 13, 950035 10.3389/fphar.2022.950035. 36249771 PMC9554617

[ref57] Charron C. E.; Russell P.; Ito K.; Lea S.; Kizawa Y.; Brindley C.; Singh D. Rv568, a Narrow-Spectrum Kinase Inhibitor with p38 MAPK-α and -γ Selectivity, Suppresses COPD Inflammation. Eur. Respir. J. 2017, 50 (4), 1700188 10.1183/13993003.00188-2017. 29074542

[ref58] Pelaia C.; Vatrella A.; Sciacqua A.; Terracciano R.; Pelaia G. Role of P38-Mitogen-Activated Protein Kinase in Copd: Pathobiological Implications and Therapeutic Perspectives. Expert Rev. Respir Med. 2020, 14 (5), 485–491. 10.1080/17476348.2020.1732821. 32077346

[ref59] Chambers R. C.; Mercer P. F. Mechanisms of Alveolar Epithelial Injury, Repair, and Fibrosis. Ann. Am. Thorac Soc. 2015, 12 (Suppl 1), S16–20. 10.1513/AnnalsATS.201410-448MG. 25830828 PMC4430974

[ref60] Jacobsen N. R.; Pojana G.; White P.; Møller P.; Cohn C. A.; Smith Korsholm K.; Vogel U.; Marcomini A.; Loft S.; Wallin H. Genotoxicity, Cytotoxicity, and Reactive Oxygen Species Induced by Single-Walled Carbon Nanotubes and C(60) Fullerenes in the Fe1-Mutatrade Markmouse Lung Epithelial Cells. Environ. Mol. Mutagen 2008, 49 (6), 476–87. 10.1002/em.20406. 18618583

[ref61] Cox G. W.; Mathieson B. J.; Gandino L.; Blasi E.; Radzioch D.; Varesio L. Heterogeneity of Hematopoietic Cells Immortalized by V-Myc/V-Raf Recombinant Retrovirus Infection of Bone Marrow or Fetal Liver. J. Natl. Cancer Inst 1989, 81 (19), 1492–6. 10.1093/jnci/81.19.1492. 2778838

[ref62] Blasi E.; Mathieson B. J.; Varesio L.; Cleveland J. L.; Borchert P. A.; Rapp U. R. Selective Immortalization of Murine Macrophages from Fresh Bone Marrow by a Raf/Myc Recombinant Murine Retrovirus. Nature 1985, 318 (6047), 667–70. 10.1038/318667a0. 4079980

[ref63] Ertürk A.; Becker K.; Jährling N.; Mauch C. P.; Hojer C. D.; Egen J. G.; Hellal F.; Bradke F.; Sheng M.; Dodt H. U. Three-Dimensional Imaging of Solvent-Cleared Organs Using 3disco. Nat. Protoc 2012, 7 (11), 1983–95. 10.1038/nprot.2012.119. 23060243

