# Supplementary material for: Nanoparticle-Exposure-Triggered Virus Reactivation Induces Lung Emphysema in Mice
Source: ACS Nano. 2023 Oct 19;17(21):21056–72. doi: 10.1021/acsnano.3c04111 (PMC10655245; doi:10.1021/acsnano.3c04111)
Supplement: Supplementary file 1 — nn3c04111_si_001.pdf [file nn3c04111_si_001.pdf]

Supporting Information

Nanoparticle exposure triggered virus reactivation induces lung emphysema in mice

Lianyong Han, Verena Haefner, Youjia Yu, Bing Han, Hongyu Ren, Martin Irmler, Johannes Beckers, Qiongliang Liu, Annette Feuchtinger, Ali Oender Yildirim, Heiko Adler, Tobias Stoeger

Detailed methods

Nanoparticle characterizations

Carbonaceous spherical nanoparticles (CNP; Printex90, Degussa, Frankfurt, Germany) and double-walled carbon nanotubes (DWCNT; Nanocyl, SA, Belgium) were used in this study. Detailed characterizations of NPs were shown in **Table S1**.

Table S1: Characterization of NPs in the study

**Nanoparticles**	**Name**	**Acronym**	**Size (nm)**	**BET (m^2/g)**	**Source**
Carbon black	Printex 90	CNP	14	272	Evonik Degussa GmbH, Germany
Double walled carbon nanotubes	DWCNT	DWCNT	10x1000	660	Nanocyl SA, Belgium

Average Size and size distribution of particle dispersions

CNP and DWCNT dispersed in water or medium and DWCNT dispersed in water

supplemented to 1% Pluronic F-127 were used in this study. The average size (Z-Ave) and size distribution (represented by the polydispersity index = PdI) was measured with photon correlation spectroscopy using a Dynamic Laser Scatter (DLS) Zetasizer Nano ZS. (Malvern Instruments Ltd., Malvern, UK).

Table S2: Average Size and size distribution

**Particle type**	**Suspended in**	**Z-Average (nm)**	**SD**	**PdI**	**SD**
CNP	water	144.933	0.802	0.207	0.008
CNP	medium	171.500	0.721	0.205	0.010
DWCNT	water	717.567	132.771	0.795	0.102
DWCNT	medium	16.393	0.023	0.430	0.006
DWCNT	1% Pluronic F-127 in water	369.533	76.883	0.570	0.047

Cell culture

Ana-1 cell line was cultured in RPMI 1640 medium (Gibco, Grand Island, NY, USA) supplemented with 15% fetal bovine serum (FBS, PAN Biotech, Aidenbach, Germany), 1% non-essential amino acids (NEAA; Gibco, Grand Island, NY, USA), 2 mM L-glutamine (Gibco, Grand Island, NY, USA), 100 U/ml Penicillin and 100 µg/ml Streptomycin (Gibco, Grand Island, NY, USA). Latently MHV-68 infected Ana-1 cells (Ana-1/MHV-68 cells) were cultured in RPMI 1640 medium supplemented with 10% FBS, 2 mM L-glutamine, 100 U/ml Penicillin, 100 µg/ml Streptomycin and 5 µg/ml Hygromycin (Merck KGaA, Darmstadt, Germany). BHK-

21 cells (ATCC: CCL-10) were grown in Glasgow-MEM medium (PAN Biotech, Aidenbach, Germany) supplemented with 5% FBS, 5% tryptose phosphate broth (Gibco, Grand Island, NY, USA), 2 mM L-glutamine, 100 U/mL Penicillin and 100 μg/mL Streptomycin. The overlay medium for plaque assay is prepared by dissolving sterile Methylcellulose in BHK-21 culture medium.

Determination of cell viability by WST-1 assay

Cell viability was measured with WST-1 assay (Roche Diagnostics, Mannheim, Germany) according to the manufacturer's instructions. Briefly, Ana-1 or Ana-1/MHV-68 cells were seeded in a density of 10,000 cells / well in a 96-well plate and incubated in 37 °C incubator for 24 h. Cells were treated with indicated concentrations in 100 μl for proper time. For some experiments, cells were pretreated with inhibitors 30 min prior to treatments. After treatment, 10 μl WST-1 reagent was added to each well and mixed thoroughly, protected from light. Then, the plate was incubated for 1 h at 37 °C. The supernatant was transferred to a microcentrifuge tube and centrifuged at 14,000 rpm for 10 min to remove NP agglomerates. 80 μl of supernatant was added to a new plate and the absorbance was measured using Microplate Reader (TECAN Group Ltd. Maennmedorf, Switzerland) at 450 nm.

LDH cytotoxicity assay

LDH assay (Roche Diagnostics, Mannheim, Germany) was used to assess the cytotoxicity of treatments by quantifying the release of lactate dehydrogenase (LDH) into medium due to cell membrane damage. Briefly, cells were seeded into a 24-well plate in a density of 200,000 cells

/ well and incubated in 37 °C incubator for 24 h. Cells were then exposed to CNP (50 μg/ml), DWCNT (50 μg/ml) or LPS (1 μg/ml) for 24 h. For some experiments, cells were pretreated with inhibitors 30 min prior to treatments. After 24 h, supernatants were collected and centrifuged at 14,000 rpm for 10 min to remove nanoparticles, then stored on ice for assay. Cells lysed with 1% Triton X-100 in equal volume medium were used as a high control. 30 μl of samples supernatant and 5 μl high control were taken and brought to 100 μl with serum-free medium. 100 μl serum-free medium was used as background control. 100 μl freshly prepared LDH working solution were added to each well. After incubation for 15 min at RT, 50 μl 1N HCl were added to stop the reaction. The absorbance was measured at a wavelength of 492 nm.

Determination of gene expression by qPCR

Whole cell RNA was isolated with NucleoSpin RNA Plus kit (MACHEREY-NAGEL, Duren, Germany) following the instructions of the manufacturer. Briefly, cells were cultured and treated in 24-well plates. After treatment, cell supernatant was collected and stored at -80 °C for further experiments. Then, 350 μl lysis buffer was added to lyse cells for 10 min at RT. Afterwards, cell lysate was transferred to genomic DNA removal column and centrifuged for 60 s at 11,000 x *g*. Then, RNA binding was adjusted followed by washing with wash buffer. Finally, RNA was eluted with 30 μl RNase-free H_2O for 60 s at 11,000 x *g*. This step was repeated one more time to get a final volume of 60 μl. RNA concentration was measured with Nanodrop (Thermo Fisher Scientific, Waltham, MA, USA). Subsequently, RNA was reverse-transcribed using superscript kit (Invitrogen, Waltham, MA, USA). Then, cDNA was used to analyze the target gene expression by real time quantitative PCR using SYBR Green PCR

master mix (Thermo Fisher Scientific, Waltham, MA, USA). For viral gene expression determination, Open Reading Frame 50 (ORF50, expressed in the lytic phase of virus life cycle) and Open Reading Frame 73 (ORF73, continuously expressed in all phases of virus life cycle) were analyzed, and 60S ribosomal protein L8 (Rpl8) was used as a housekeeping gene. The primer pairs used in this study are shown in **Table S3**. The fold change in expression is shown as $2^{-\Delta\Delta Ct}$, where $\Delta Ct = Ct_{\text{target gene}} - Ct_{\text{housekeeping gene}}$, and $\Delta\Delta Ct$ reflects ΔCt of each treatment normalized to control. Thus, the mean value for control is 1, and the value for treatment is shown as the fold change compared to control. Results of three or four independent experiments are shown.

Table S3: Primer pairs used in this study

**Gene name**	**Forward primer (5'-3')**	**Reverse primer (3'-5')**
MHV-68 ORF50	GGAATTTCTGCAGCGATGGCCTCT	CCTCTTTTGTTTCAGCAGAGACTCCA
MHV-68 ORF73	CTGGACTCCTCATCACCTT	TGTCTGAGCGTCTTCCAC
Rpl8	CAGTGAATATCGGCAATGTTTTG	TTCACTCGAGTCTTCTTGGTCTC
Hmox1	TTCTGGTATGGGCCTCACTGG	ACCTCGTGGAGACGCTTTACA
Ptgs2	CAACACCTGAGCGGTTAC	GTTCCAGGAGGATGGAGT
Il1b	CAACCAACAAGTGATATTCTCCATG	GATCCACACTCTCCAGCTGCA
Il6	TAGTCCTTCCTACCCCAATTTCC	TTGGTCCTTAGCCACTCCTTC
Tnf	CACCACGCTCTTCTGTCT	GGCTACAGGCTTGTCACTC

Saa3	GAAGCTGGTCAAGGGTCT	GTCAGCTCTTGAGTCCTCTG
Nos2	CCTGTGAGACCTTTGATG	CCTATATTGCTGTGGCTC
Actb	TCCATCATGAAGTGTGACGT	GAGCAATGATCTTGATCTTCAT
Hprt	GTTGGATACAGGCCAGACTTTGT	CACAGGACTAGAACACCTGC

In vitro lytic virus protein detection by immunofluorescence staining

NPs treated Ana-1/MHV-68 cells after 24 h were stained with polyclonal rabbit serum again lytic protein of MHV-68 to investigate the lytic virus protein expression induced by different NPs.[1] Ana-1/MHV-68 cells were seeded on sterile cover slides in 24-well plates and cultured for 24 h. Cells were treated with CNP (50 µg/ml), DWCNT (50 µg/ml), LPS (1 µg/ml) or left untreated for 24 h. The supernatant was removed and 300 µl cold 4% Paraformaldehyde (PFA) solution was added into each well to fix cells for 15 min at RT. Solution was discarded and cells were washed two times with PBS followed by quenching with 50 mM NH_4Cl solution in PBS for 10 min at RT. Then, cells were permeabilized and blocked with 1% Triton X-100, 5% BSA in PBS for 60 min at RT. After blocking, cells were incubated with primary antibody diluted in 1% Triton X-100, 5% BSA in PBS overnight at 4 °C. On the second day, cells were washed two times with PBS and incubated with fluorescence-labeled secondary antibody (Goat anti-Rabbit IgG Alexa Fluor 555, Thermo Fisher Scientific, Waltham, MA, USA) and Alexa Fluor 488 Phalloidin (Thermo Fisher Scientific, Waltham, MA, USA) in 1% Triton X-100, 5% BSA in PBS for 60 min at RT, with the plate protected from light. After removal of the solution, cells were incubated with DAPI (Merk KGaA, Darmstadt, Germany) in PBS for 10 min at RT, with the plate protected from light. After washing in PBS, cells were mounted with DAKO

mounting medium (Agilent Technologies, Santa Clara, CA, USA) on microscope slides. Slides were dried at RT and stored at 4 °C. Images were taken with Leica Laser Scanning Confocal fluorescence microscope (SP5-II, Leica, Wetzlar, Germany)

Flow cytometry

The expression of CD11b and CD11c (CD11b anti-human/mouse, PE, 130-113-235; CD11c anti-human/mouse, FITC, 130-110-837; Miltenyi Biotec. Bergisch Gladbach, Germany) on Ana-1 cells were detected by flow cytometry. Briefly, Ana-1 cells were collected after cultivation and centrifuged at 400 g for 5 min to get a pellet. Then the pellet was washed with FACS buffer and centrifuged at 300 g for 10 min at 4 °C. The pellet was resuspended in a density of $10*10^6$ cells/ml in FACS buffer and transfer to FACS tubes.

Cells were incubated with CD11b-PE, CD11c-FITC antibody (1:50 dilution) on ice for 15 min, protect cells from light. Afterwards, cells were washed with 1 ml FACS buffer and centrifuged at 300 g for 5 min at 4 °C. The supernatant was discarded and cells were resuspended with 200 µl FACS buffer. Cells were ready for flow cytometry analysis.

Cells were acquired by FACScantoTM II flow cytometer and analyzed by FlowJo software (BD Biosciences, San Jose, CA, USA). Mean fluorescence Index (MFI) was used to show the intensity of CD11b or CD11c expression of the cells.

Western blot

Whole cell protein from NPs treated Ana-1/MHV-68 cells was extracted with RIPA buffer (contains 50 mM Tris, 150 mM NaCl, 1 mM EDTA, 0.5% (w/v) deoxycholic acid, 0.1% (w/v)

SDS, 0.5% (v/v) Nonidet P-40, pH 8.0) supplemented with protease and phosphatase inhibitor cocktail (Thermo Fisher Scientific, Waltham, MA, USA). Protein concentration was measured with BCA kit (Thermo Fisher Scientific, Waltham, MA, USA) following the instructions of the manufacturer. All protein samples were adjusted to equal concentrations. 20 to 40 μg protein was denatured and loaded onto SDS-PAGE gel. Pre-stained precision protein ladder (Bio-Rad, Hercules, CA, USA) was used for evaluation of electrophoresis and protein size. Electrophoresis was run at 100 V for 10 min and 120 V at least 60 min followed by blotting to PVDF membrane (Bio-Rad, Hercules, CA, USA) at 100 V for at least 60 min. Then, membrane was blocked in Roti block solution (Carl Roth, Karlsruhe, Germany) for 60 min at RT. Primary antibodies were diluted in Roti block solution and incubated with membrane overnight at 4 °C. On the second day, membrane was incubated in Goat anti-Rabbit secondary antibody (#7074; Cell Signaling Technology, Danvers, MA, USA) or Goat anti-Mouse secondary antibody (RPN4201; GE Healthcare, Chicago, IL, USA) diluted in Roti block solution. After washing, membrane was incubated with ECL Western Blotting Detection Reagents (GE Healthcare, Chicago, IL, USA), and bands were detected with ChemiDoc MP Imaging system (Bio-Rad, Feldkirchen, Germany) and analyzed with Quantity One Software (Bio-Rad, Feldkirchen, Germany). p-p38 (Thr180/Tyr182) (#4511), p-ERK1/2 (Thr202/Tyr204) (#4370), p-JNK (Thr183/Tyr185) (#4668), ERK1/2 (#4695), p-MAPKAPK2 (Thr334) (#3007), MAPKAPK2 (#3042) were purchased from Cell Signaling Technology, Danvers, MA, USA; p38α/β (sc-7972) and JNK (sc-7345) were purchased from Santa Cruz Biotechnology; β-actin (A3854) was purchased from Merck KGaA, Darmstadt, Germany.

Transcriptomic study by microarray analysis

Total RNA was isolated followed by the same procedure described above. The Agilent 2100 Bioanalyzer was used to assess RNA quality and only high quality RNA (RIN > 7) was used for microarray analysis. Total RNA was amplified using the WT PLUS Reagent Kit (Thermo Fisher Scientific Inc., Waltham, MA, USA). Amplified cDNA was hybridized on Mouse Clariom S arrays (Thermo Fisher Scientific, Waltham, MA, USA). Staining and scanning (GeneChip Scanner 3000 7G) was done according to manufacturer's instructions. The Transcriptome Analysis Console (TAC; version 4.0.1.36; Thermo Fisher Scientific, Waltham, MA, USA) was used for quality control and to obtain annotated normalized SST-RMA gene-level data. Statistical analyses were performed by utilizing the statistical programming environment R (v4.0.4). Genewise testing for differential expression was done employing the limma t-test and regulated gene sets were defined by raw p-value < 0.05. To reduce background, gene sets were filtered using DABG p-value < 0.05 in more than half of the samples in at least one group per comparison. Heatmaps were done in R with pheatmap package. Pathway analysis were generated through the use of Gene Set Enrichment Analysis (GSEA) software (v4.1.0, Broad Institute, Cambridge, MA, USA) and clusterProfiler package in R (version 4.0.4), comparing the Gene Ontology Biological Process (GOBP) database.[2-3] Dotplot, barplot and treeplot were used to visualize enriched terms according to the use of ggplot2 package. Array data has been submitted to the GEO database at NCBI (GSE223818).

Reactive oxygen species (ROS) detection

Intracellular ROS of Ana-1 cells were measured with DCFH-DA probe (Merck, Darmstadt,

Germany). Briefly, Ana-1 cells were seeded in black 96-well plates and incubated for 24 h at 37 °C. Medium was removed and fresh medium containing 30 µM DCFH-DA was added in half volume and incubated for 30 min in the incubator. NACA (5 mM; Merck, Darmstadt, Germany) was added together with DCFH-DA. After 30 min, cells were treated with NPs (50 µg/ml) or H_2O_2 (10 mM) in half volume containing 2 times concentration, equal volume medium was added as untreated group. Fluorescence with excitation / emission at 485 nm / 535 nm was measured after 10, 30 and 60 min.

Multiplex Cytokine/Chemokine Analysis

Cytokines and chemokines in bronchoalveolar lavage fluid were measured using the multiplex bead array system Bio-Plex Pro Mouse Chemokine Assay Panel 31-Plex (#12009159, Bio-Rad Laboratories GmbH), following the manufacturer's instructions. Data were acquired using the Luminex200 system with BioPlex Manager 6.1 software. Standard curves were fitted using the logistic-5PL regression type.

Bronchoalveolar lavage (BAL) preparation and cell differentiation

After 24 h of CNP instillation, mice were anesthetized with midazolam/medetomidine/fentanyl (MMF). Immediately after sacrification, BAL was performed by cannulating the trachea and infusing the lungs six times with 1.0 ml PBS without calcium and magnesium, as described previously.[4] The first two times lavage were collected separately and centrifuged at 425 x *g* for 20 min at 4 °C to separate fluids for cytokine detection. The pellet was also collected with the rest lavage to finally get all cells. The pellets were suspended in RPMI 1640 medium (Gibco,

Grand Island, NY, USA) supplemented with 10% fetal bovine serum (FBS, PAN Biotech, Aidenbach, Germany), total living cell number was counted with trypan blue dye and 30,000 cells were used for each cytospin. Cytospin slides were stained with May-Grünwald- Giemsa solution followed by macrophages, neutrophils and lymphocytes number counting.

Immunohistochemistry (IHC) staining

Lytic virus protein expression in mouse lungs was detected by immunohistochemistry (IHC) staining. Briefly, mouse lung tissue was fixed in 4% PFA at 4 °C and washed with PBS two times. Then, lung tissue was embedded in paraffin and cut to 3 μm slices. Deparaffinization and rehydration were done by dipping slices into Xylene, 100% Ethanol, 90% Ethanol, 80% Ethanol, 70% Ethanol in order and rinsed with distilled H_2O. Slices were then incubated in 1.8% H_2O_2-Methanol solution for 20 min to block peroxidase and rinsed with distilled H_2O, followed by heat-induced epitope retrieval (HIER) with Citrate pH = 6.0 buffer. Later, slices were incubated with 1 x blocking buffer (Rodent Block M; Biocare Medical/Zytomed Systems, Berlin, Germany) at RT for at least 30 min and incubated with a polyclonal rabbit serum directed against lytic proteins of MHV-68 (describe here[1]; 1:2000 dilution) at 4 °C overnight. After washing, Rabbit-on-rodent-AP-polymer (Biocare Medical/Zytomed Systems, Berlin, Germany) was added to each slice and incubated for 20 min. After washing, Vulcan Fast Red Chromogen Kit (Biocare Medical/Zytomed Systems, Berlin, Germany) was used to show the presence of alkaline phosphatase (AP) enzyme. Slices were later counterstained with Haematoxylin followed by 2 times Xylene incubation. Finally, slices were mounted with Entellan solution and dried under the chemical hood for at least 1 h before imaging. Design-based stereology was

used to analyse IHC slides using a light microscope (Olympus BX51) equipped with a computer-assisted stereological toolbox (newCAST, Visiopharm) running Visiopharm Integrator System (VIS) software (v.6.0.0.1765).

Immunofluorescence (IF) staining and ZEISS scanning

Anti MHV-68 immunofluorescence double staining together with macrophage markers CD11b and CD11c were performed and used further for ZEISS scanning.

Lung tissue slices were deparaffinized and rehydrated by dipping slices into Xylene, 100% Ethanol, 90% Ethanol, 80% Ethanol, 70% Ethanol in order and rinsed with distilled H_2O. Heat-induced epitope retrieval (HIER) was done by incubation of slices in Citrate pH = 6.0 buffer. Later, slices were incubated in blocking solution (5% goat serum + 0.03% Triton X-100 in PBS) at RT for 1 h, followed by incubation with a primary antibody (polyclonal rabbit serum directed against lytic proteins of MHV-68, described before [1], CD11b (ab133357; Abcam, Cambridge, UK), CD11c (#97585, Cell Signaling Technology, Danvers, MA, USA) in an antibody diluent (AD; 1% BSA + 0.03% Triton X-100 in PBS) at 4 °C overnight. After washing, fluorescence-labeled secondary antibody (Goat anti-Rabbit IgG Alexa Fluor 555, A21428, Thermo Fisher Scientific, Waltham, MA USA; Goat anti-Rabbit IgG Alexa Fluor 647, A21245, Thermo Fisher Scientific, Waltham, MA, USA), F-actin dye (Alexa Fluor 488 Phalloidin, A12379, Thermo Fisher Scientific, Waltham, MA, USA) and DAPI (D9564; Roche, Basel, Switzerland) incubation were performed in antibody diluent at RT for 1 h, with the sides protected from light. After washing, slices were mounted with Dako fluorescence mounting medium (Agilent Technologies, Santa Clara, CA, USA). Images were taken either by LEICA confocal

fluorescence microscope (SP5-II, Leica, Wetzlar, Germany) for high resolution images or Zeiss Axio Scan.Z1 (Carl Zeiss, Jena, Germany) for slices scanning. Later analysis was performed with ZEN Blue 2.3 software. For BAL cells cytospin staining, cytospin slides were firstly fixed with 4% PFA for 15 min at RT and washed two times with PBS, followed by incubation with primary antibodies and secondary antibodies as usual.

TUNEL assay and quantification

DNA fragmentation and cell death of lung tissue was assessed using the terminal deoxynucleotidyl transferase dUTP nick end labelling (TUNEL) assay according to manufacturer’s instruction (ab66110, Abcam, Cambridge, Massachusetts) on paraffin-embedded sections. dUTP-labeled DNA was visualized directly using Olympus BX51 fluorescence microscopy. Nuclei were counterstained with DAPI. TUNEL staining was quantified by taking 6 random fields of view per mouse lung, using an Olympus BX51 fluorescence microscope. TUNEL positive cells were counted and normalized to the DAPI events for each field. 3 mice per group were analyzed were the mean of the 6 fields per lung indicated at the individual dots in the graph.

Quantification of interstitial inflammation score and MCL

Airspace enlargement (MCL) and inflammation score was assessed by hematoxylin and eosin (H&E) staining. Design-based stereology was used to analyze sections using an Olympus BX51 light microscope equipped with a computer-assisted stereological toolbox (CAST) software Visopharm Integrator System (VIS) v6.0.0.1765 (newCAST, Visiopharm) on H&E stained

lung slides for assessing the airspace enlargement and the tissue inflammation volume. Air space enlargement was assessed by quantifying mean chord length (MCL) on 30 field of view per lung, a line grid was superimposed on lung section images taken with the ×20 objective. Intercepts of lines with alveolar septa and points hitting airspace were counted to calculate MCL applying the formula $MCL = \sum P_{air} \times L(p)/\sum I_{septa} \times 0.5$. P_{air} are the points of the grid hitting air spaces, L(p) is the line length per point, and I_{septa} is the sum of intercepts of alveolar septa with grid lines. To quantify interstitial inflammation in the lung, a line grid was superimposed on lung section images taken with the ×20 objective and were analyzed on lung sections across at least 30 random fields per lung. Intercepts of lines crossing with airways and vessels were counted to calculate the interstitial inflammation $(\mu m^3/\mu m^2) = \sum P_{inflamed\ area} \times L(p)/\sum I_{intercept(A+V)}$. $P_{inflamed\ area}$ are the points of the grid hitting inflammatory tissue area, L(p) is the line length per point, and $I_{intercept(A+V)}$ is the sum of intercepts of airways and vessels to normalize the inflammatory area.

Light-sheet fluorescence (LSFM) microscopy

Mouse lungs were perfused and fixed with 4% PFA at 4°C overnight and then washed with PBS for two times. Lung lobes were blocked and permeabilized in PBSGT (0.2% gelatin, 0.5% TritonX-100 in PBS, 0.01% thimerosal) for 3 days with rotation at RT. Then, lobes were incubated with primary antibodies in PBSGT containing 0.1% saponin (10 µg/mL) for 7 days with rotation at RT. Lobes were later rinsed in washing buffer (PBS + 0.5% Triton X-100) 6 times during 1 day at RT with rotation. Then, lobes were incubated with secondary antibodies in PBSGT containing 0.1% saponin (10 µg/mL) at RT for 3 days. Secondary antibody solution

was passed through 0.22 µm filter to avoid secondary antibody precipitates in solution. Lobes were rinsed again in washing buffer 6 times during 1 day at RT with rotation. Then, lobes were incubated in 50% Tetrahydrofuran (THF) in H_2O overnight subsequently with 50%, 80% and 100% THF for 1 h. Next, lobes were incubated in 100% THF overnight and 100% THF 1 h. Then, lobes were incubated in Dichloromethane (DCM) for 20 to 40 min followed by incubation in Dibenzyl Ether (DBE) solution for at least 4 to 6 h until imaging. Images were taken with UltraMicroscope II (Miltenyi Biotec, Bergisch Gladbach, Germany).

Supporting figures & legends

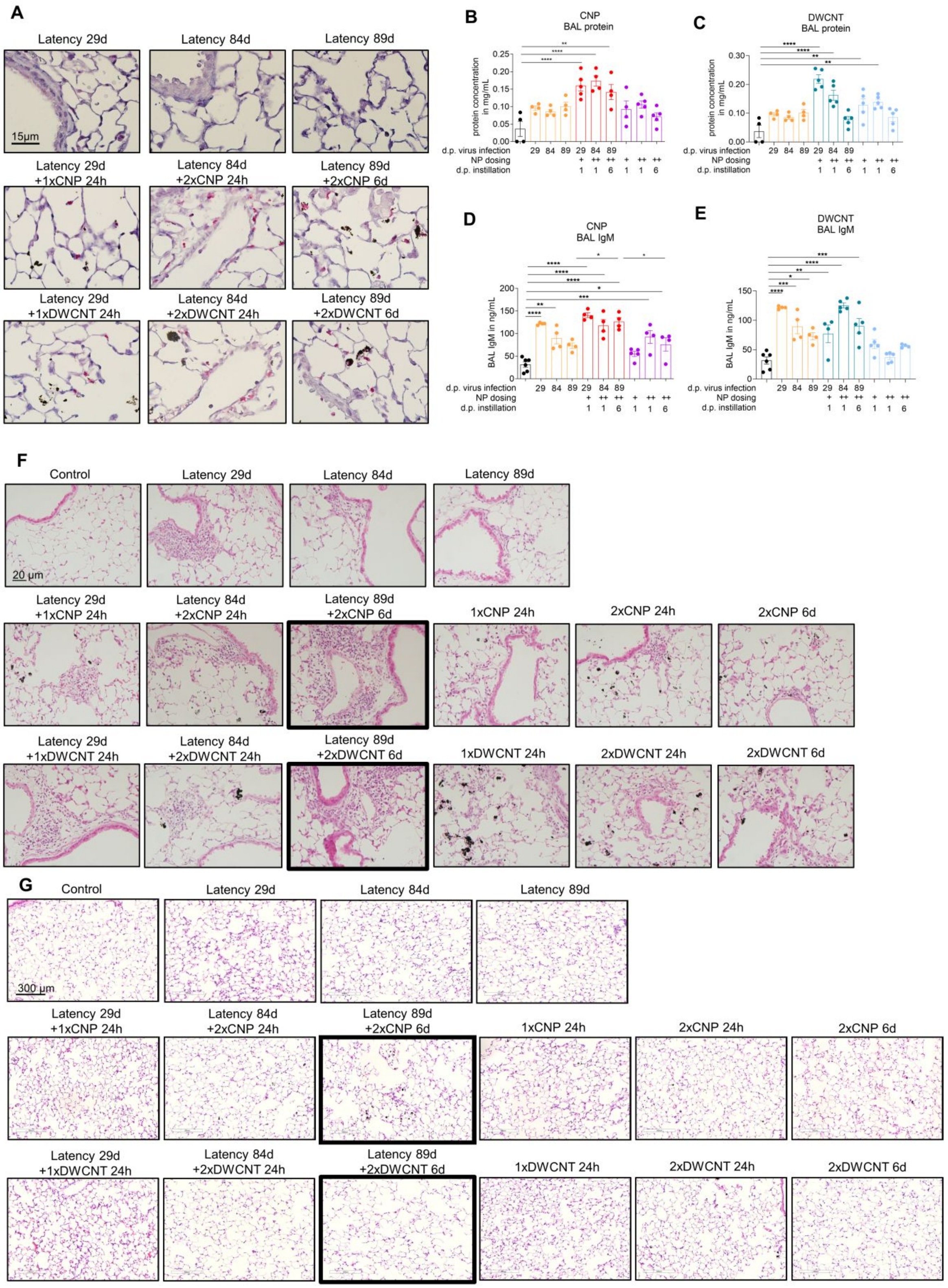

Figure S1. Repeated NP exposure reactivates MHV-68 and increases interstitial inflammation, alveolar air space and epithelial-endothelial barrier disruption

(A) Overview of IHC detection of MHV-68 lytic protein expression during latency and reactivation, induced by 1st and 2nd NP exposure during virus latency. Lung slides were stained with anti-MHV-68 serum, detected by Vulcan fast red and counterstained with Hematoxylin. MHV-68 lytic protein detection shows virus reactivation (red) after 1st and 2nd NP exposure in latently infected mice. Scale bar: 15 µm. **(B-E)** Bronchoalveolar lavage (BAL) was performed to investigate BAL protein and IgM levels as signs of alveolar barrier injury. **(B)** Measurement of BAL protein concentration with BCA assay shows significant increases in latent virus infected and CNP instilled mice (N = 4-5). **(C)** Measurement of BAL protein concentration with BCA assay shows significant increases in latent virus infected and non-infected mice 1st and 2nd exposure to DWCNT after 24h (N = 4-5). **(D)** BAL IgM levels assessed via IgM ELISA shows elevated levels 6 days after the 2nd CNP dosing in comparison to latency of CNP only (N = 4-5). **(E)** BAL IgM levels assessed via IgM ELISA shows elevated levels in latency and 1st and 2nd DWCNT exposure in virus latency (N = 4-5). **(F & G)** Interstitial inflammation analyzed in H&E stained lung tissue samples, at various conditions of latency (29d, 84d and 89d after infection), after 1st and 2nd NP (CNP or DWCNT) exposure during virus latency, and 24h after the 1st and 2nd NP treatment and additionally 6 days after the 2nd NP instillation. **(F)** H&E stained lung tissue shows increased interstitial inflammation in lungs of mice 89 days after MHV-68 infection (latency) and 6 days after 2nd CNP or DWCNT instillation. Scale bar: 20 µm. The bold framed images are shown in Figure 1.D **(G)** H&E stained lung tissue shows increased air space in lung tissue of mice 89 days after MHV-68 infection and 6 days after 2nd CNP or DWCNT instillation. Scale bar: 300 µm.

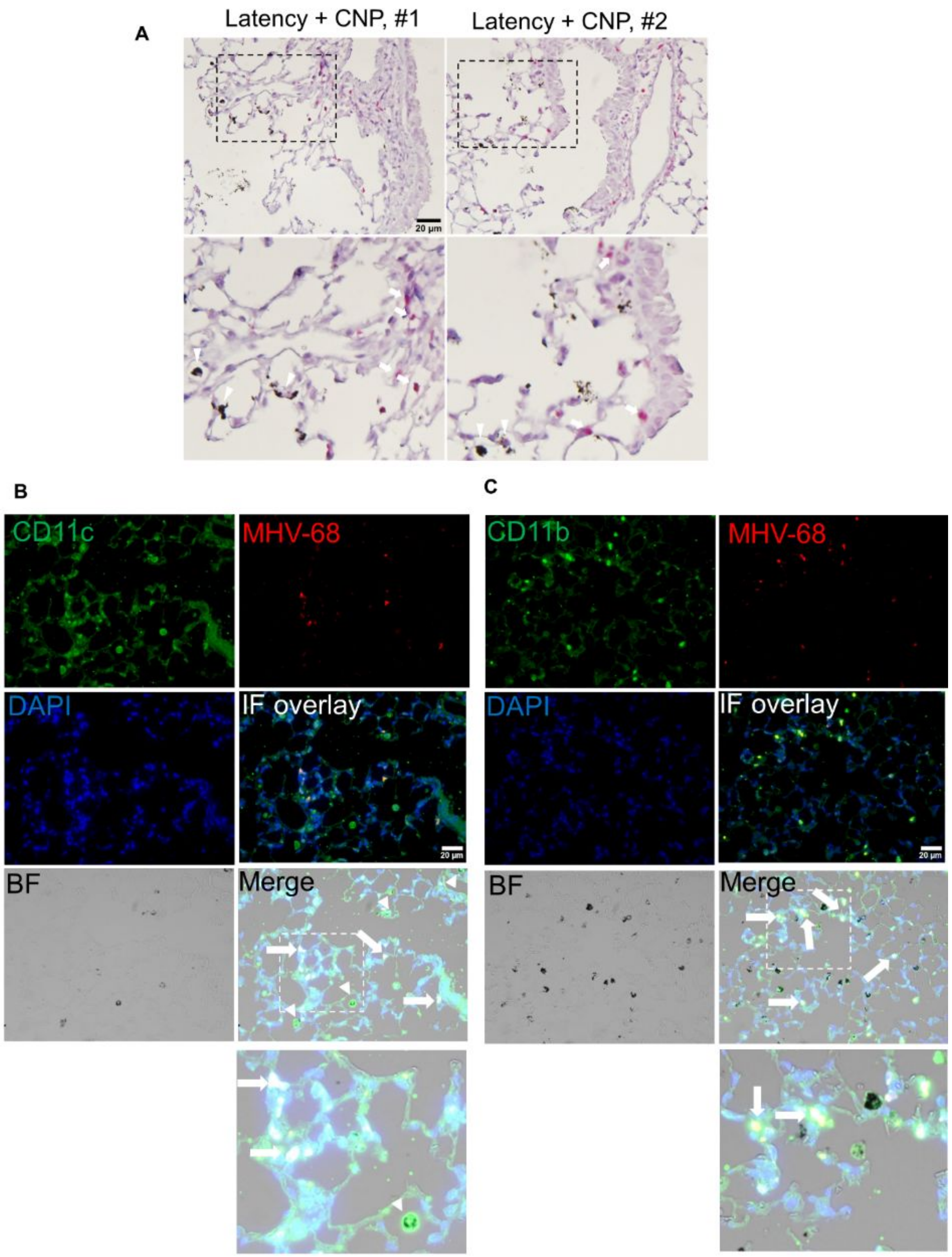

Figure S2. MHV-68 reactivating macrophages show no nanoparticle agglomerates laden

(A) IHC staining of MHV-68 lytic protein localization and nanoparticle distribution in mouse lung tissue after CNP exposure 24 h during latency. Mouse lung sections were stained with

anti-MHV-68 serum, detected by Vulcan fast red and counterstained with Hematoxylin. Red: anti-MHV-68, Black: nanoparticle agglomerates. Scale bar: 20 μm. **(B)** IF double staining and bright field imaging show the relationship between CD11c positive alveolar macrophages, MHV-68 lytic protein and CNP agglomerates laden. Arrows on merge image represent MHV-68 reactivation macrophages and triangles represent alveolar macrophages having phagocytosed CNP agglomerates. Representative images of CD11c (Green), anti-MHV-68 (Red), DAPI (Blue), IF overlay, BF (bright field) and all channel merge are shown. Scale bar: 20 μm. **(C)** The relationship between CD11b positive macrophages, MHV-68 lytic protein and CNP agglomerates laden. Arrows on merge image represent MHV-68 reactivation macrophages. Representative images of CD11b (Green), anti-MHV-68 (Red), DAPI (Blue), IF overlay, BF (bright field) and all channels merge are shown. Scale bar: 20 μm.

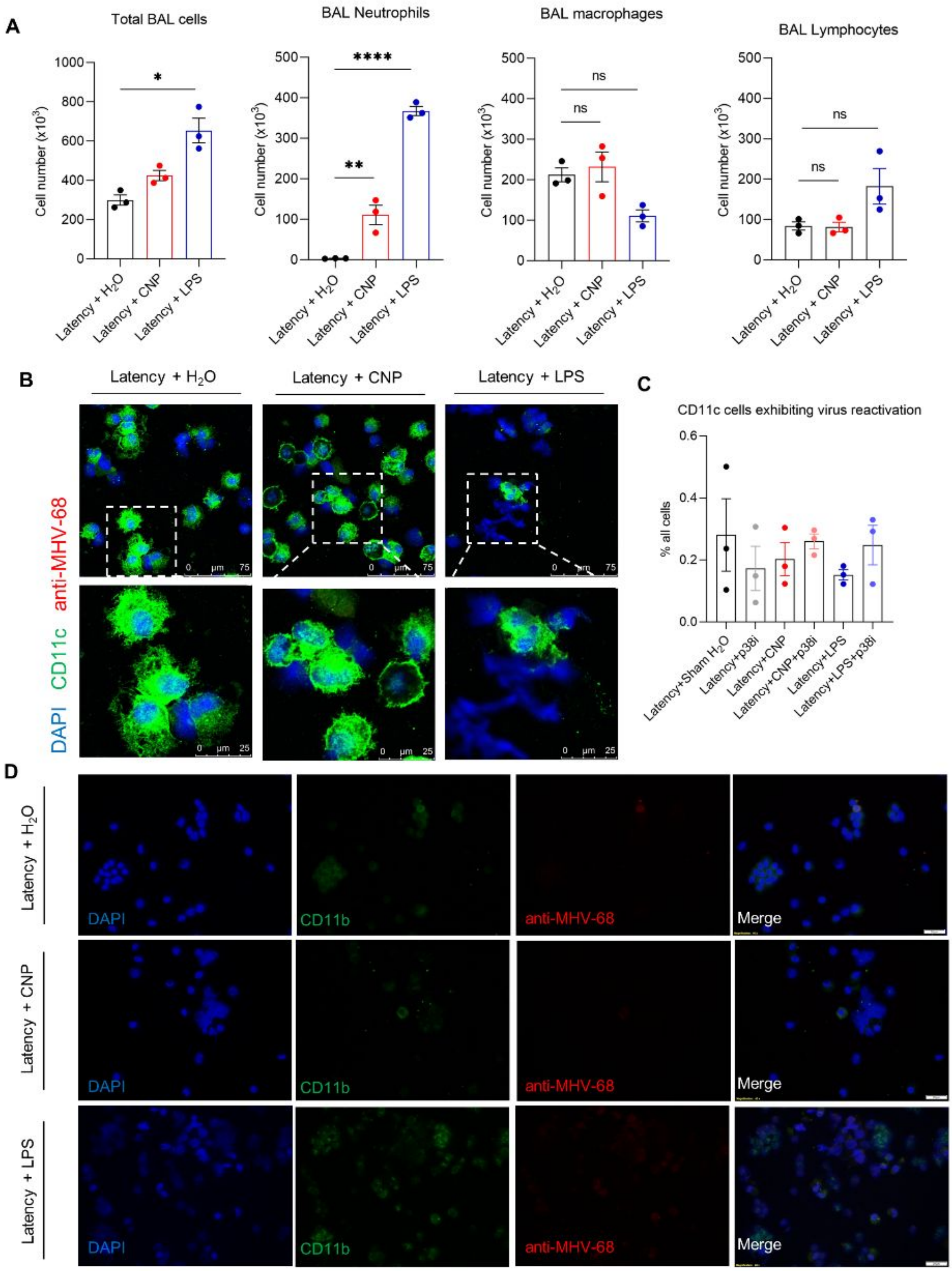

Figure S3. Alveolar macrophages but not recruited macrophages are main components in bronchoalveolar lavage (BAL) cells and hardly showed MHV-68 reactivation

(A) Total BAL cell number and cell differentiation including macrophages, neutrophils and

lymphocytes are shown. BAL cells were harvested and counted. Cytospins were performed with 30,000 cells. Cytospin slides were performed with Giemsa staining to differentiate macrophages, neutrophils as well as lymphocytes. **(B)** IF staining of BAL cells showed that the majority of macrophages after lavage are CD11c positive cells. Slides were stained with anti-MHV-68 serum (red), CD11c (green), and DAPI (blue). Images were taken with confocal fluorescence microscope (Leica, TSC-SP5-II, Wetzlar, Germany) to show detailed localization of MHV-68 reactivation. Scale bar: 250 μm (25 μm for enlarged images). **(C)** Quantification of MHV-68 positive events in CD11c stained cells in mouse lungs (N=3). Values were shown as mean ± SEM. Data were analyzed by one-way ANOVA. **(D)** IF staining of BAL cells hardly showed the appearance of CD11b positive cells. Slides were stained with anti-MHV-68 serum (red), CD11b (green), and DAPI (blue). Images were taken with Olympus BX-51 fluorescence microscope (Olympus, Hamburg, Germany) to show detailed localization of MHV-68 reactivation. Scale bar: 20 μm.

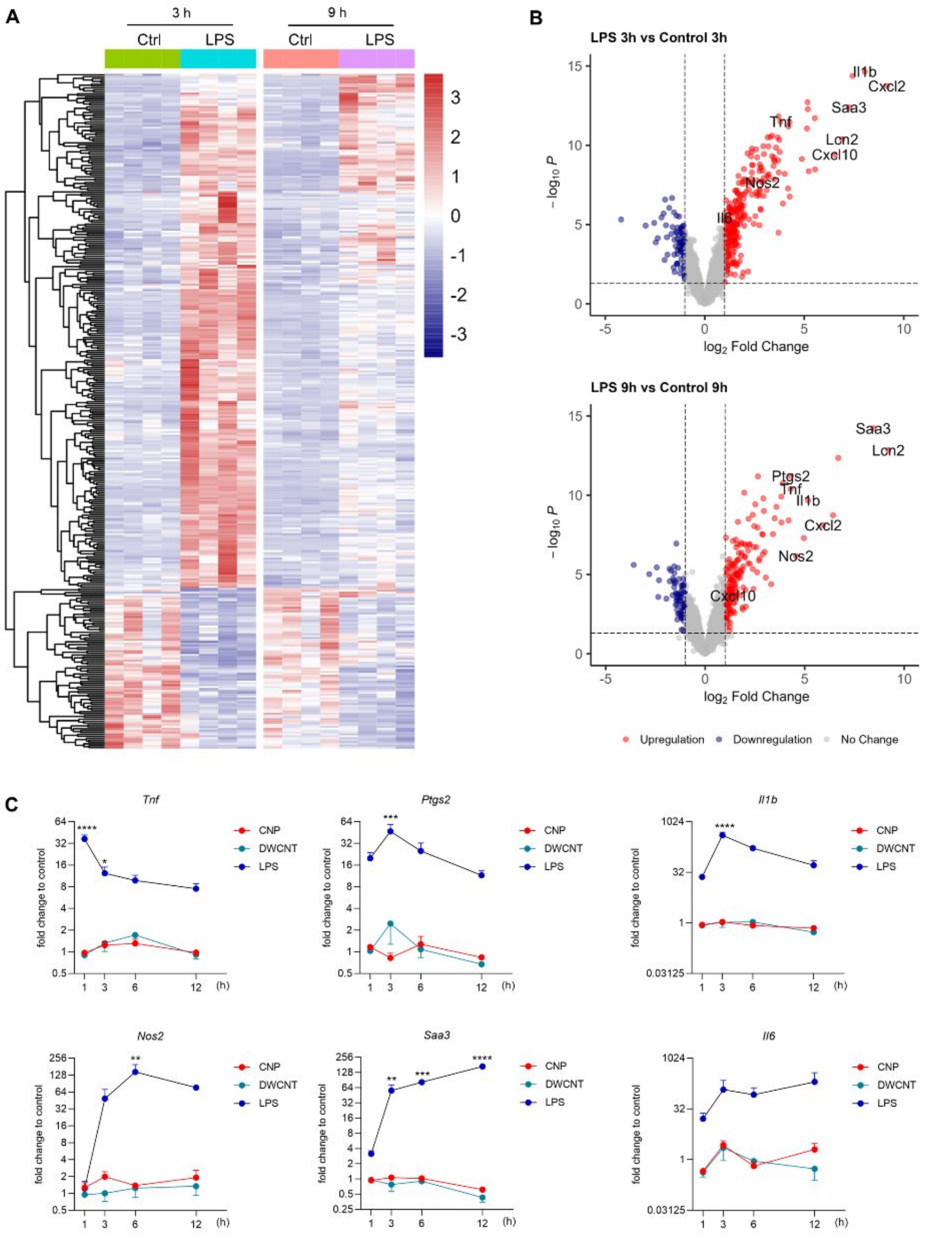

Figure S4. A strong pro-inflammatory response was induced by LPS exposure

Top regulated genes induced by LPS (1µg/ml) were sorted and a heatmap was generated with R software (version 4.0.4) and is shown in **(A)**. Volcano plots of " 'LPS 3 h' vs 'Control 3 h' "

and " 'LPS 9 h' vs 'Control 9 h' " are shown in **(B)**. Classical pro-inflammatory response genes, *e.g.* *Il1b*, *Saa3*, *Nos2* and *Tnf*, were significantly upregulated. **(C)** Confirmation of classical pro-inflammatory genes expression by qPCR. Ana-1/MHV-68 cells were exposed to CNP, DWCNT and LPS, and an equal amount of medium as control. The expression of mouse genes *Tnf, Saa3*, *Il1b*, *Nos2*, *Ptgs2* and *Il6* was measured by qPCR after 1, 3, 6 and 12 h. Fold changes of genes were calculated and are shown. Four or five independent experiments were performed and included for statistical analysis. Data were analyzed by Student's *t* test, "*" indicates statistically significant difference to "control". *: $P < 0.05$, **: $P < 0.01$.

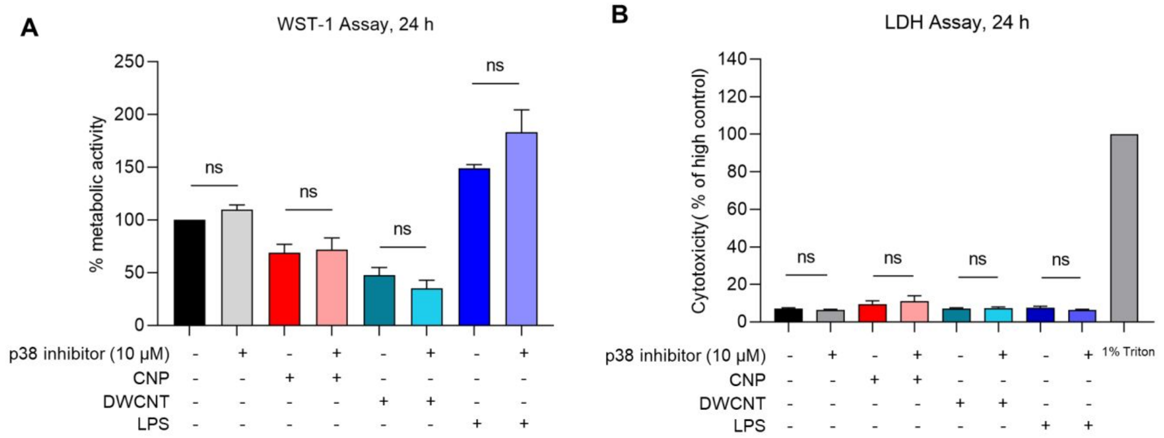

Figure S5. p38 inhibition showed no further toxicity to Ana-1/MHV-68 cells

Ana-1/MHV-68 cells were treated with specific p38 inhibitor (10 µM) 30 min prior to CNP (50 µg/ml), DWCNT (50 µg/ml) or LPS (1µg/ml), or an equal amount of medium as control. Cytotoxicity of p38 inhibitor was determined by WST-1 assay **(A)** and LDH assay **(B)** after 24 h. For LDH assay, 1% Triton X-100 lysed cell supernatant was used as a positive control for LDH release. Three or four independent experiments were performed and included for statistical analysis. Data were analyzed by Student's *t* test, "*" indicates statistically significant difference to "control". *: $P < 0.05$.

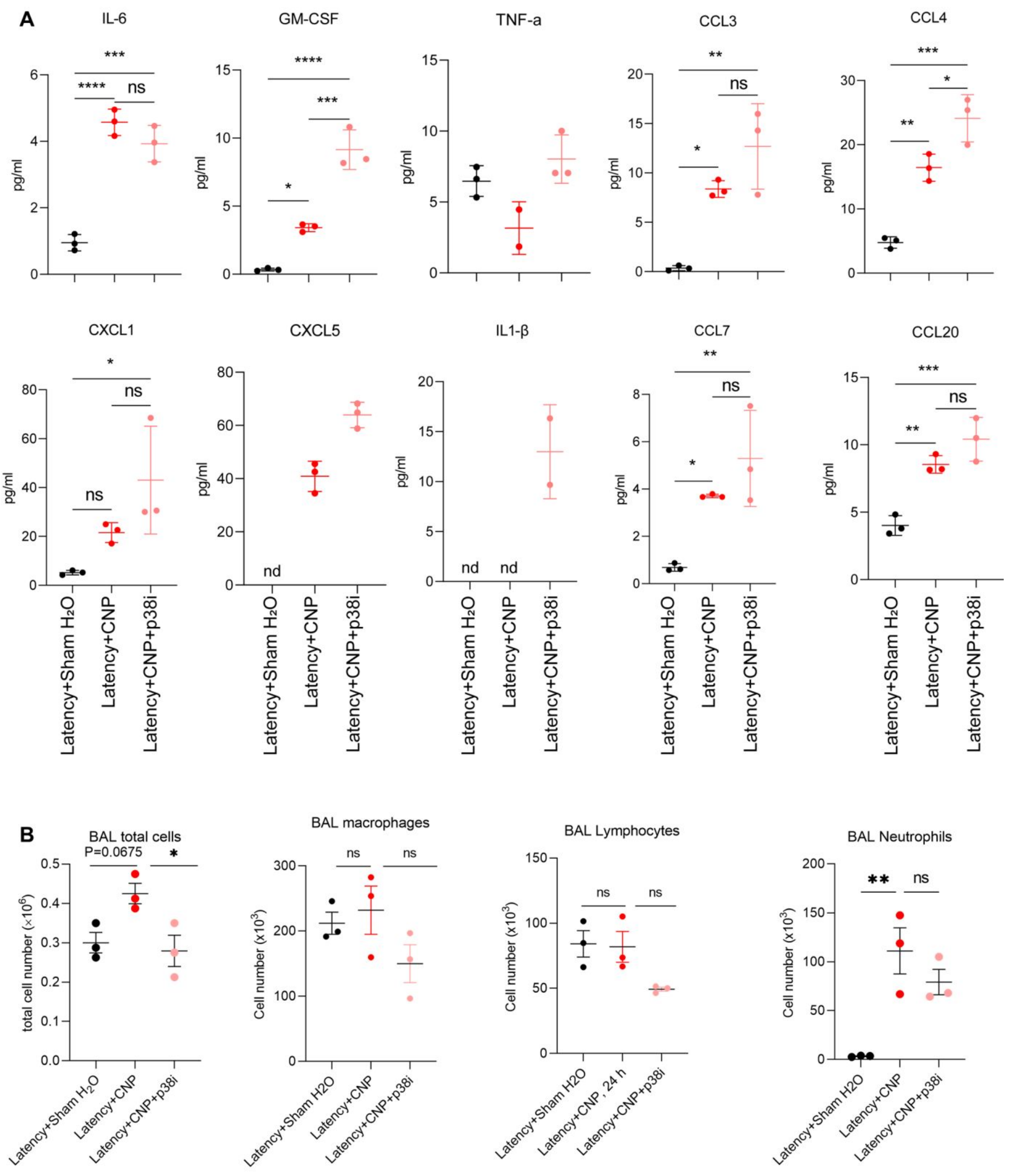

Figure S6. CNP exposure caused airspace inflammation and is not attenuated by p38 inhibition.

(A) 31-plex assay was performed to measure cytokine release into BAL fluid. Major cytokines which were released upon CNP exposure during latency are shown. (B) The effect of p38 inhibition on BAL total cell number, macrophages, lymphocytes and neutrophils are shown.

Values are shown as mean ± SEM (N=3). Data were analyzed by one-way ANOVA followed by Tukey's multiple comparisons test. *: $P < 0.05$. **: $P < 0.01$. ***: $P < 0.001$. ***: $P < 0.0001$. ns: no significance. nd: not detected.

Abbreviations

AIF-1: allograft inflammatory factor 1

BAL: Bronchoalveolar Lavage

BF: Bright field

BSA: Bovine serum albumin

CAST: computer-assisted stereological toolbox

CLD: Chronic lung disease

CMV: Cytomegalovirus

CNP: Carbonaceous nanoparticle

CNT: Carbon nanotubes

COPD: Chronic obstructive pulmonary disease

d: day

DC: Dendritic cell

DCFH-DA: 2',7'-Dichlorodihydrofluorescein diacetate

DWCNT: Double-walled carbon nanotube

EBV: Epstein-Barr virus

ELISA: Enzyme-linked immunosorbent assay

FACS: Fluorescence-activated single cell sorting

GOBP: Gene Ontology Biological Process

GPNMB: glucoprotein nmb

GSEA: Gene Set Enrichment Analysis

HHV6: Human Herpesvirus 6

HHV8: Human Herpesvirus 8

HIER: Heat-Induced Epitope Retrieval

H_2O_2: Hydrogen peroxide

IF: Immunofluorescence

IHC: Immunohistochemistry

IPF: Idiopathic pulmonary fibrosis

IVC: individually ventilated cages

KSHV: Kaposi's sarcoma-associated herpesvirus

LDH: Lactate Dehydrogenase

LPS: Lipopolysaccharide

LSFM: Light-sheet fluorescence microscopy

MAPK: Mitogen-activated protein kinase

MCL: Mean chord length

MHV-68: Murine gammaherpesvirus 68

NACA: N-acetylcysteine amide

NAC: N-acetyl-l-cysteine

NP: Nanoparticle

PFA: Paraformaldehyde

PFU: Plaque-forming unit

PM: Particulate matter

qPCR: Quantitative polymerase chain reaction

ROS: Reactive Oxygen Species

RT: Room temperature

RTA: replication and transcriptional activator

SEM: Standard error of mean

SiO_2: Silicon dioxide

TiO2: Titanium dioxide

TUNEL: Terminal deoxynucleotidyl transferase dUTP nick end labeling

WHO: World Health Organization

References

1. Steer, B.; Adler, B.; Jonjic, S.; Stewart, J. P.; Adler, H., A Gammaherpesvirus Complement Regulatory Protein Promotes Initiation of Infection by Activation of Protein Kinase Akt/Pkb. *PLoS One* **2010,** *5* (7), e11672.
2. Yu, G.; Wang, L. G.; Han, Y.; He, Q. Y., Clusterprofiler: An R Package for Comparing Biological Themes among Gene Clusters. *Omics* **2012,** *16* (5), 284-7.
3. Wu, T.; Hu, E.; Xu, S.; Chen, M.; Guo, P.; Dai, Z.; Feng, T.; Zhou, L.; Tang, W.; Zhan, L.; Fu, X.; Liu, S.; Bo, X.; Yu, G., Clusterprofiler 4.0: A Universal Enrichment Tool for Interpreting Omics Data. *Innovation (Camb)* **2021,** *2* (3), 100141.
4. Sattler, C.; Moritz, F.; Chen, S.; Steer, B.; Kutschke, D.; Irmler, M.; Beckers, J.; Eickelberg, O.; Schmitt-Kopplin, P.; Adler, H.; Stoeger, T., Nanoparticle Exposure Reactivates Latent Herpesvirus and Restores a Signature of Acute Infection. *Part Fibre Toxicol* **2017,** *14* (1), 2.
